# Testing drivers of acoustic divergence in cicadas (Cicadidae: *Tettigettalna*)

**DOI:** 10.1111/jeb.14133

**Published:** 2022-12-13

**Authors:** Raquel Mendes, Vera L. Nunes, Eduardo Marabuto, Gonçalo J. Costa, Sara E. Silva, Octávio S. Paulo, Paula C. Simões

**Affiliations:** ^1^ Centre for Ecology, Evolution and Environmental Changes (cE3c), Departamento de Biologia Animal, Faculdade de Ciências Universidade de Lisboa, Campo Grande Lisboa Portugal

**Keywords:** acoustic signals, cicada, ecological selection, evolution, phylogenetic comparative analysis

## Abstract

Divergence in acoustic signals may have a crucial role in the speciation process of animals that rely on sound for intra‐specific recognition and mate attraction. The acoustic adaptation hypothesis (AAH) postulates that signals should diverge according to the physical properties of the signalling environment. To be efficient, signals should maximize transmission and decrease degradation. To test which drivers of divergence exert the most influence in a speciose group of insects, we used a phylogenetic approach to the evolution of acoustic signals in the cicada genus *Tettigettalna*, investigating the relationship between acoustic traits (and their mode of evolution) and body size, climate and micro‐/macro‐habitat usage. Different traits showed different evolutionary paths. While acoustic divergence was generally independent of phylogenetic history, some temporal variables’ divergence was associated with genetic drift. We found support for ecological adaptation at the temporal but not the spectral level. Temporal patterns are correlated with micro‐ and macro‐habitat usage and temperature stochasticity in ways that run against the AAH predictions, degrading signals more easily. These traits are likely to have evolved as an anti‐predator strategy in conspicuous environments and low‐density populations. Our results support a role of ecological selection, not excluding a likely role of sexual selection in the evolution of *Tettigettalna* calling songs, which should be further investigated in an integrative approach.

## Graphical abstract

Habitat influence in acoustic divergence.

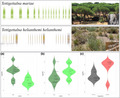

## 1 INTRODUCTION

Understanding how new species arise has been an all‐time goal of evolutionary biology, where the divergence in particularly relevant characters may trigger the speciation process. Many taxa rely on acoustic signalling for interspecific recognition and mate attraction, which confers these signals an important role in speciation (Wilkins et al., 2013). Acoustic signals can evolve rapidly, facilitating the detection of morphologically cryptic forms (Marshall & Cooley, 2000), which albeit acoustically distinct, likely represent an early stage in the speciation continuum (e.g. Alexander, 1962; Marshall et al., 2008).

Acoustic signals are subject to different evolutionary pressures. Sexual selection, environmental conditions, morphological constraints and the presence of predators and/or parasitoids are just some of the factors that may act concomitantly or in opposition to each other (Endler, 1992; Wilkins et al., 2013). As a result of its influence on the divergence of sexual traits—which can lead to reproductive isolation—sexual selection has been regarded as an important engine of speciation (e.g. Butlin et al., 2012; West‐Eberhard, 1983). However, recent studies show that the combination of both natural and sexual selection is particularly powerful to initiate and complete speciation (Oneal & Knowles, 2013; van Doorn et al., 2009). For instance, song frequencies differ with body size, since in many animals there is a negative correlation between body length and dominant song frequency (Bradbury & Vehrencamp, 1998). This allometric relationship has also been demonstrated for cicadas, with sound frequencies seemingly being constrained by body size (Bennet‐Clark & Young, 1994). Hence, ecological selection on body size may promote acoustic divergence and vice versa (Wilkins et al., 2013).

The acoustic adaptation hypothesis (AAH) postulates that acoustic signals diverge according to environmental physical properties that influence the attenuation and degradation of a signal during transmission through the atmosphere to maximize signal transmission and decrease signal degradation (Wiley & Richards, 1978, 1982). Consequently, animal sounds appear to adapt to a specific habitat in both frequency and temporal components (e.g. Badyaev & Leaf, 1997; Morton, 1975). Microclimate and vegetation structure are important in sound degradation due to atmospheric absorption and scattering, respectively. Sound attenuation increases with increasing temperature and decreasing humidity, while high‐frequency sounds attenuate faster and are more scattered by foliage than low‐frequency sounds (Morton, 1975; Wiley & Richards, 1982). Animals in hotter and drier microclimates should therefore produce lower frequency sounds than animals in more temperate or humid climates. Signals of wood warblers and bats were found to diverge along microclimate gradients due to climate influence in atmospheric absorption (Snell‐Rood, 2012). Divergence was primarily at the spectral level, but bats also changed signal duration between seasons. Temporal and spectral attributes vary with vegetation structure because scattering increases positively with habitat closeness. Consequently, songs in closed habitats are predicted to have a longer duration, lower repetition rate and lower frequencies, with shorter notes and longer intervals, than songs in open habitats (Badyaev & Leaf, 1997; Ey & Fischer, 2009). While acoustic adaptation has been substantially studied in vertebrates (mammals, birds and anurans) with inconsistent results (review in Ey & Fischer, 2009), studies with insects are scarce and lend little support (e.g. Couldridge & van Staaden, 2004). To investigate acoustic adaptation hypotheses in insects, one should control for phylogenetic history, and body size, and use populations of a species or closely related species occupying different habitats (Balakrishnan, 2016), which in most cases has not been available.

**FIGURE 1:**
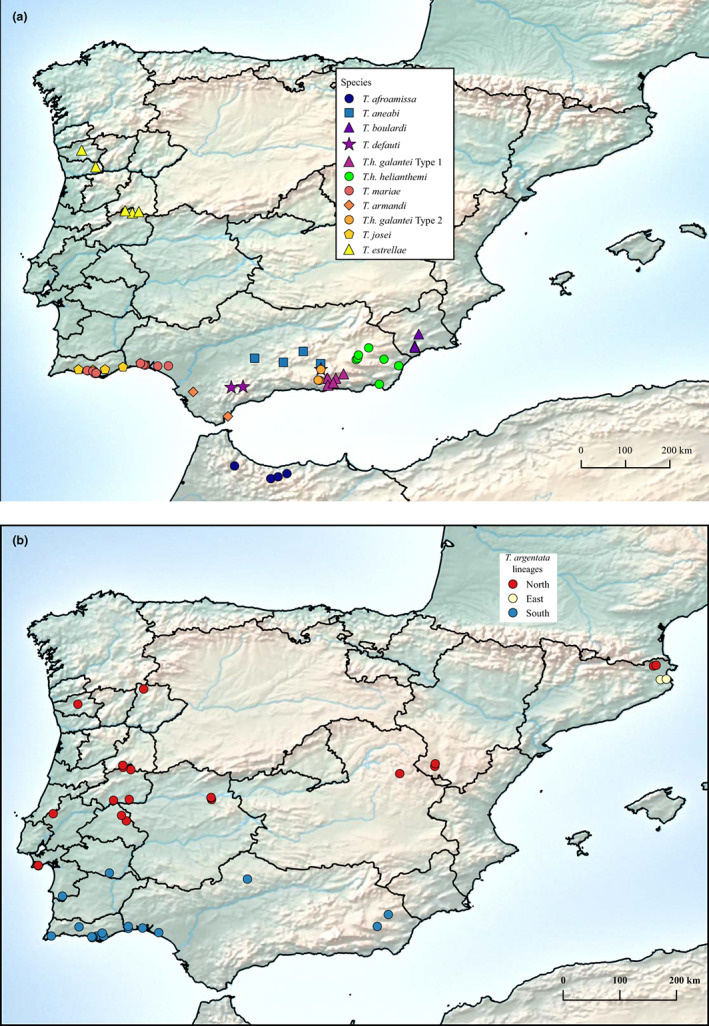
Sampling locations. (a) Sampling locations for 11 *Tettigettalna* species. (b) Sampling locations of *T. argentata* lineages.

Males of *Tettigettalna* produce species‐specific calling songs to attract females and are morphologically very similar. Sound is the main character used for species identification and is thought to play an important role in their reproductive isolation preventing hybridization among sibling species (Simões & Quartau, 2006). Calling songs have a simple acoustic construction with no harmonic structure. Echemes are the main units of the calling song, grouping in species‐specific rhythm arrangements that constitute phrases (Puissant & Sueur, 2010). *Tettigettalna* species exist only in the West Mediterranean Basin and occupy a variety of habitats. Most species are restricted to the Iberian Peninsula (see Figure 1) and are likely to represent a recent radiation (Costa, 2017). The current Iberian climate is very heterogeneous, influenced by both the Atlantic Ocean and the Mediterranean Sea, with North‐South and East‐West precipitation gradients, with increased precipitation towards North and West, while temperatures increase along a North‐South gradient (Cunha et al., 2011; Rivas‐Martínez, 2005). Following the Köppen‐Geiger climatic classification, the peninsula is divided into two major climatic regions: (1) temperate with dry or temperate summer in the northeast, the west coast of Portugal, and the mountain regions and (2) temperate with dry or hot summers, covering most of the southern central plateau region and the Mediterranean coastal regions (Cunha et al., 2011). Additionally, local orographic and geological conditions allow for the formation of microclimates, from hot desert climate (e.g. Almeria, Murcia) to cold with temperate and dry summer climate found in the highest altitudes of the Sierra Nevada (Cunha et al., 2011). *Tettigettalna argentata* is the only species with a wide distribution (Gogala & Gogala, 1999; Hertach, 2008; Puissant & Sueur, 2010). This species is geographically structured into three or four genetically distinctive lineages (Costa, 2017; Nunes et al., 2014). The remaining species occupy the south of the peninsula in a parapatric, patchwork‐like pattern, except *T. estrellae*, whose distribution is limited to the North of Portugal (Sueur et al., 2004), and *T. afroamissa* which is endemic to the north of Morocco in the Rif and Middle Atlas Mountains (Costa et al., 2017). Nevertheless, several species' distribution ranges overlap.

Most species do not show host specialization and are observed in a broad diversity of plants within favourable habitats (Puissant & Sueur, 2010); however, some species are more often found in closed, arboreal vegetation while others almost exclusively inhabit open, shrubby or herbaceous vegetation types. The extent to which vegetation structure (open vs. closed) and local climate are of importance to the acoustic diversification of these species remains to be discovered.

Here, we explore the relationship between calling song variation and ecological divergence in *Tettigettalna* species, while accounting for body size and phylogeny, through the employment of phylogenetic comparative methods (PCM). These methods are an analytical toolkit used to study species in a historical framework to understand the mechanism behind their diversification (Garamszegi, 2014). Two types of data are required (1) an accurate phylogeny of the taxon under study and (2) interspecific phenotypic data for the same taxon (Garamszegi, 2014). Previous studies have successfully applied PCM to the study of acoustic diversification in vertebrates, such as frogs (e.g. Escalona Sulbarán et al., 2019; Goutte et al., 2016) and birds (e.g. Derryberry et al., 2012; Gonzalez‐Voyer et al., 2013). We specifically test predictions from the AAH, using climate variables as a proxy for microclimate gradients and macro and microhabitat structure as a proxy for vegetation openness. Additionally, we test the phylogenetic signal and mode of evolution of acoustic variables.

Phylogenetic signal measures the degree to which a trait value is correlated to the phylogeny, with estimates increasing when trait similarity among closely related species increases. A phylogenetic signal is frequently equated with an underlying evolutionary process. High‐phylogenetic signal has been construed as evolutionary conservatism (e.g. Ossi & Kamilar, 2006) or gradual evolution through time (e.g. Brownian motion) and is commonly connected with genetic drift, stabilizing selection or physiological constraints (e.g. Blomberg & Garland, 2002; Wiens et al., 2010). Low phylogenetic signal has been interpreted as evolutionary ‘lability’ (e.g. Blomberg et al., 2003) or high evolutionary rates and is usually associated with adaptive radiations, divergent selection, or convergent evolution. Inferring the evolutionary process from phylogenetic signal estimation, however, may be tricky and prone to misjudgements (Revell et al., 2008). Nevertheless, in conjunction with other analyses, it can provide insights into the underlying processes driving a particular trait evolution.

Our main goal is to investigate the relative role of stochastic (e.g. genetic drift) and deterministic (e.g. ecological selection) processes in the divergence and evolution of the acoustic signals produced by 12 species of cicadas in a variety of microclimates and micro‐habitats. If environmental acoustic properties are a key factor in the acoustic divergence of *Tettigettalna* species, then patterns of acoustic divergence should correlate with the physical properties of the habitat. Specifically, (1) songs in closed habitats should be longer, with shorter notes and long intervals, and have lower frequencies than songs in open habitats and/or, (2) songs in hot, dry climates should have lower frequencies than songs in temperate, humid climates. Alternatively, finding a correlation between acoustic and ecological divergence in ways contrary to the AAH predictions together with a low phylogenetic signal may indicate divergence by ecological selection driven by factors other than the maximization of song transmission. If, on the other hand, acoustic divergence is mainly driven by neutral, stochastic processes, we expect to find no correlation between acoustic and ecological divergence and a high‐phylogenetic signal. It is important to note that different song attributes may have different evolutionary histories.

## 2 MATERIALS AND METHODS

### 2.1 Sampling and acoustic data

Sampling took place in Portugal, Spain and Morocco (Figure 1; Table 1) during the summer seasons (June to August) from 2011 to 2017. *Tettigettalna* species have a parapatric distribution and some species have a very restricted range. Our sampling reflects this, with species with broader distributions being more heavily sampled and species with narrower distributions having smaller sample sizes (Table 1).

**TABLE 1:** Sampling information.

Species	Sites	Songs	Sample individuals (N)			Average ± SE songs per individual
			Morphologic analysis	Acoustic analysis	Total	
*T. afroamissa*	4	125	9	5	11	25 ± 18.1
*T. aneabi*	4	298	11	7	16	42.6 ± 21.6
*T. argentata* East	2	9	10	3	13	3 ± 1.0
*T. argentata* North	19	213	39	40	67	5.3 ± 4.1
*T. argentata* South	16	251	57	54	92	4.7 ± 2.5
*T. boulardi*	3	18	5	8	13	2.3 ± 1.3
*T. defauti*	3	70	5	5	11	7.8 ± 5.0
*T. estrellae*	5	152	5	22	27	6.9 ± 2.8
*T. h. galantei* Type 1	6	34	19	15	34	2.3 ± 1.2
*T. h. galantei* Type 2	3	24	12	11	18	2.2 ± 1.4
*T. h. helianthemi*	7	59	20	18	37	3.3 ± 2.3
*T. josei*	6	83	5	22	27	3.8 ± 2.9
*T. armandi*	2	38	5	3	8	12.7 ± 8.7
*T. mariae*	12	184	40	40	70	4.6 ± 2.4
Total	92	1558	242	255	44	–

*Note*: Sites: number of locations sample per species; songs: total number of songs analysed per species. Morphologic analysis: number of individual males used in morphological analysis; acoustic analysis: number of individual males used in acoustic analysis; total: total number of males sampled (not necessarily the sum of the former). Average ± SE songs/ind – average plus standard‐error number of songs per individual male for each species.

All specimens were males and species identification were carried out in the field according to their calling songs. Male calling songs were field recorded using a Marantz PMD 661 Portable SD recorder (20 Hz – 24 kHz) connected to a Telinga Pro 7 Dat‐mic (60 Hz – 18 kHz) microphone (Twin Science). Several minutes were recorded for each male, usually encompassing several consecutive songs. GPS coordinates of all sampling sites were registered with a Garmin Oregon 550t.

Acoustic recordings were initially visualized and inspected for quality in Audacity version 2.1.0 (Audacity Team, 1999–2015). For each record, individual songs were identified according to the known description of each species' calling song (Costa, 2017; Puissant & Sueur, 2010; Sueur et al., 2004). Each individual song was then saved as a separate file. When necessary, the noise between echemes that could potentially interfere with echeme detection was manually removed. Recordings with background noise that could not be separated from the cicada sounds were discarded. Each song was then analysed with the R package *warbleR* version 1.1.5 (Araya‐Salas & Smith‐Vidaurre, 2016) for automatic determination of temporal coordinates (start and end time of each echeme), applying an absolute amplitude envelope and a Fast Fourier Transformation size of 512. The function also outputs a spectrogram of the recording marking the temporal coordinates of each echeme, which was used to assess echeme detection accuracy. Songs in which echeme detection was not accurate were discarded. For each echeme, dominant frequency values were extracted as a 12‐point time series using the *dfreq* function in the R package *warbleR* with a Hanning window and Fast Fourier Transformation size of 512. Echemes, where a 12‐point time series could not be measured, were excluded from the analysis.

The final data set then comprised 1558 calling songs from a total of 255 individuals from 14 OTUs (operational taxonomic units), which included all the known *Tettigettalna* species and three *T*.

*argentata* lineages. Henceforward, OTUs will be referred to as species, for simplicity. Detailed information for each male cicada used is detailed in Supplemental Information Table S1. Since all the analyses were performed at the species level, we calculated species averages from individual averages: call duration (CD; in seconds), number of echemes per call (NE), echeme rate (ER; echemes per second), echeme duration (ED), interval duration (ID) and dominant frequency (DF). These values are shown in Table S2.

### 2.2 Morphologic and ecological data

As a proxy for body size, we measured pronotum length of several specimens per taxon (min.–max. = 5–57 individuals). The pronota were photographed under a Zeiss LUMAR stereoscope coupled with a TIS DFK 5MPixel camera and measures were taken in ImageJ (version 1.48v). We chose to work with pronotum length, since total body size exceeded camera focal length, and the two measures are also highly correlated (unpublished data).

The acoustic adaptation hypothesis predicts that acoustic signals evolve to reduce degradation in accordance with general climate (temperature, humidity), calling site and closeness/openness of the habitat (Endler, 1992; Ey & Fischer, 2009; Morton, 1975). To test each one of these hypotheses, we used bioclimatic variables to summarize climatic conditions potentially important in sound propagation. We used WorldClim to obtain 19 bioclimatic variables with a spatial resolution of ~1 km, covering the 1950–2000 time period (Hijmans et al., 2005). Climatic values for each population's GPS point were extracted with R and the mean value of each bioclimatic variable was calculated across the geographic range of each species. Since these variables are often auto‐correlated and to decrease the number of models calculated, we excluded the variables that correlated with the higher number of other variables according to a Pearson correlation threshold of 0.75.

To analyse the relationship between acoustic variation and habitat usage, we tested two variables chosen to represent habitat openness. First, as a measure of habitat openness at a microhabitat level, we classified the predominant calling site in a binary trait as either arboreal or shrubby/herbaceous according to field observations. For that effect, we calculated the percentage of individuals in each species calling from each substrate in our database. Most species are opportunistic in relation to calling site, which may vary with microhabitat and available vegetation. As such, this classification reflects the most common vegetation substrate in which each species could be heard calling. Second, as a proxy for habitat openness at a macrohabitat level, we used the Normalized Difference Vegetation Index (NDVI). NDVI is calculated from the visible and near‐infrared light reflected by vegetation—a measure of vegetation greenness. NDVI values range from −1.0 to +1.0. Very low NDVI values (≤0.1) indicate areas of barren rock, sand or snow; moderate values (~0.2 to 0.5) indicate sparse vegetation such as shrubs and grasslands and high NDVI values (~0.6 to 0.9) correspond to dense vegetation such as that found in temperate and tropical forests or crops at their peak growth stage (NCAR, 2018). We used the latest version of the third generation NDVI data set (NDVI3g.v1, Pinzon & Tucker, 2014) from National Oceanic and Atmospheric Administration's Advanced Very High‐Resolution Radiometer sensors (NAOO AVHRR), from the Global Inventory Modelling and Mapping Studies (GIMMS), provided by the National Aeronautics and Space Administration (NASA). This version provides half‐month NDVI values from 1981 to 2015 at a resolution of ~8 km. We used the R package *gimms* version 1.1.3 (Detsch, 2020) to download a subset of the data set comprising the years 2010–2015 (roughly the time span of our sampling) since the NDVI data set does not have data after 2015. Using the same package, data were rasterized with quality control, by discarding all pixels with flag values of 1 or 2 (spline‐interpolation and snow/cloud cover respectively). To speed up computation, this step was carried out by employing an extent for the geographic area of interest (Portugal, Spain, and Morocco). We subsequently calculated monthly maximum‐value composites (MVC) from the half‐monthly data sets. This procedure retains only the highest monthly NDVI value for each pixel location. We used the R package *raster* version 3.3.13 (Hijmans, 2020) to extract the monthly NDVI values for each GPS coordinate in our data set. We then calculated the mean NDVI values for each species. Since adult cicadas are only active in the summer months (June – August) only these months were used in this calculation. NDVI values were dichotomized as high and low, using as a cut‐off point the average value between the maximum and minimum species value. This was done to facilitate the interpretation of results from the two habitat variables (NDVI and calling site) and to allow the use of NDVI as different habit regimes in the model fitting analysis (see section 2.6).

### 2.3 Phylogeny

**FIGURE 2:**
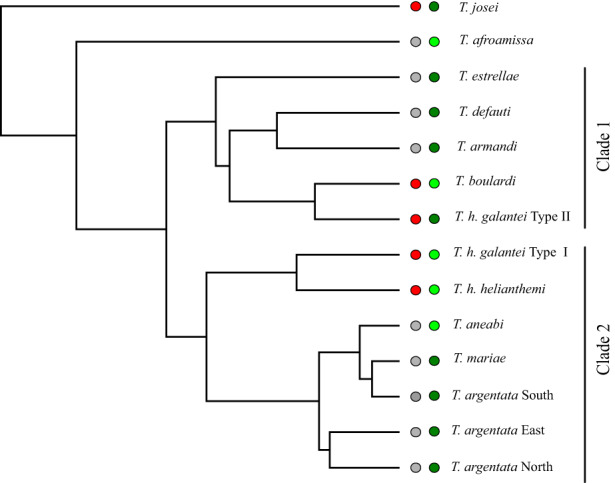
*Tettigettalna* species tree from Costa (2017). Dots refer to habitat characterization. Red dots: Shrubby species; grey dots: Arboreal species; dark grey dots: High NDVI; light green dots: low NDVI.

We obtained the most recent molecular phylogeny based on the concatenation method from Costa, 2017. This phylogeny includes all known species and identifies some genetically divergent lineages, which we used as species in our analyses (Figure 2). Briefly, sequences from three mitochondrial loci, COI‐Lep (650 bp); COI‐CTL (850 bp) and ATP (800 bp), alongside the nuclear locus, EF‐1α (600 bp) were used. Two samples per species or population (of *T. argentata*) were selected, each corresponding to the most ancestral and most recently derived haplotypes (defined previously by preliminary maximum likelihood and Bayesian inference trees). Three outgroups were used, *Cicada barbara*, *Cicada orni* and *Hilaphura varipes*. For our analysis, we trimmed the original tree by removing species/specimens using the ‘drop. tip’ function in the R package ape (Paradis & Schliep, 2019).

### 2.4 Phylogenetic signal

We tested the presence of phylogenetic signal in the morphometric, ecological and acoustic variables. For the continuous variables (all acoustic and morphometric variables), we estimated the evolutionary parameter Pagel's lambda (λ; Pagel, 1999), using the *phylosig* function in the R package *phytools* version 0.7.47 (Revell, 2012). We chose Pagels's lambda over other indices as it proved to be the least affected by the number of species in the phylogeny (Münkemüller et al., 2012). Pagel's λ follows a Brownian motion (BM) model of trait evolution, which assumes that phenotype traits evolve through a series of continuous, random, independent steps and variance between species accumulates in proportion to shared phylogenetic history (Felsenstein, 1985). λ normally varies between 0 (no phylogenetic signal) and 1 (high phylogenetic signal). A value of lambda equal to 0 indicates that traits covary less than expected under BM (i.e. independently of phylogenetic history), whereas a value of lambda equal to 1 indicates that traits covary as expected under BM (i.e. closely related species are more similar to each other).

For the binary habitat variables (calling site and NDVI), we estimated the D parameter (Fritz & Purvis, 2010), using the *phylo.d* function in the R package *caper* version 1.0.1 (Orme et al., 2018). A value of D equal to 0 indicates a high phylogenetic signal and a value of *D* equal to 1 indicates no phylogenetic signal. The value of *D* can be both smaller than 0 (highly conserved) and >1 (overdispersed) (Fritz & Purvis, 2010).

### 2.5 Correlated evolution of calling song

The evolutionary association between each acoustic variable and the morphological and ecological traits was analysed using phylogenetic generalized least squares analyses (PGLS; Martins & Hansen, 1997). PGLS is an extension of the generalized least squares method, that accounts for nonindependent species data by incorporating a phylogenetic covariance matrix in the error structure of the model. The expected covariance matrix is proportional to the amount of shared evolutionary history between the species in a given phylogeny (assuming a BM model of evolution) and likelihood methods can be applied to find transformations of the matrix that improve the fit of the model. The most used transformation is the λ transformation. When λ = 1, the model assumes a BM model of evolution, while values of λ < 1 indicate that the covariation between the traits is lower than expected under Brownian evolution (Symonds & Blomberg, 2014). We used the function *pgls* in the R package *caper* with λ transformation to test each hypothesis of correlated evolution, separately. Temporal acoustic variables and pronotum length were log‐transformed, and dominant frequency was square‐transformed to meet the statistical requirements of normality and homogeneity of the model's residuals (Freckleton, 2009). Bioclimatic variables were not transformed. For each model, we inspected the distribution of the residuals to assess their normality and homogeneity (Freckleton, 2009).

### 2.6 Mode of acoustic evolution

To visualize evolutionary patterns in multivariate phenotypic (acoustic) space, we plotted a ‘phylomorphospace’, which superimposes a phylogenetic tree into a subspace defined by the principal components of phenotypic variation. Phylomorphospace is a common tool used in the study of macroevolutionary processes in morphological change (Adams & Collyer, 2019) and here we extended its use to acoustic space. We used the *phylomorphospace* function in the R package *phytools* to project the phylogeny into acoustic space, ancestral states of the internal nodes were estimated by maximum likelihood. Since dominant frequency did not show to be important between species and for an easier interpretation, only the temporal acoustic variables were used (see section 3).

To discern the evolutionary processes that might influence the diversification of *Tettigettalna* acoustic signals, we assessed the fit of seven evolutionary models to our acoustic variables. We fitted three single‐rate models and four multi‐regime models to our data. For the single‐rate models, we considered: Brownian‐Motion (BM), single‐optimum Ornstein‐Uhlenbeck (OU) and Early‐Burst (EB) models. The BM model (Felsenstein, 1985) describes trait evolution as a random walk wherein the covariance structure is defined by the phylogeny (i.e. closely related species are more similar) and can be described with two parameters, the evolutionary rate parameter (σ^2^) and the mean starting value of the trait (z(0)). This model is commonly associated with evolution by neutral drift; however, a BM pattern of evolution can arise by other processes such as selection in a quickly fluctuating environment (Hansen & Martins, 1996). The OU model (Butler & King, 2004; Hansen, 1997) is an extension of the BM model with a ‘rubber band effect’ (constricted evolution) modelled by an additional parameter alpha (α), that describes the strength of attraction towards an optimal value (θ). This model is often used to model stabilizing selection. The EB model (Harmon et al., 2010) is used to model adaptive radiation, where the rate of evolution increases or decreases exponentially through time. For the multi‐regime model, we fitted a BM model (BMS, O'Meara et al., 2006) with different rate parameters (σ^2^) for each calling site discrete selection regimes (shrubby vs. arboreal) and a similar model for the NDVI discrete selection regimes (high vs. low). These models can be interpreted as directional selection (e.g. Catalán et al., 2019). Finally, we fitted two OU models with different state means and different rate parameters for those same regimes (OUMV), which model stabilizing selection. Marginal ancestral state estimates for each internal node of the tree were calculated through maximum likelihood with the function *rerootingMethod* in *phytools* and selective regimes painted in the phylogeny prior to model fitting. To account for intra‐specific variance, all models were fitted with the standard error calculated from each species' data. All single‐rate models were fitted using the function *fitContinuous* in the R package *geiger* (Pennel et al., 2014) and the multi‐regime models with the function *ouwie* from the package *OUwie* version 2.2. (Beaulieu & O'Meara, 2020). Model fit was assessed using Akaike's Information Criterion corrected for small sample sizes (AICc) and Akaike weights (AICw). An ΔAICc threshold of 4 was used to infer strong support (Burnham & Anderson, 2002).

## 3 RESULTS

### 3.1 Sampling and habitat characterization

**FIGURE 3:**
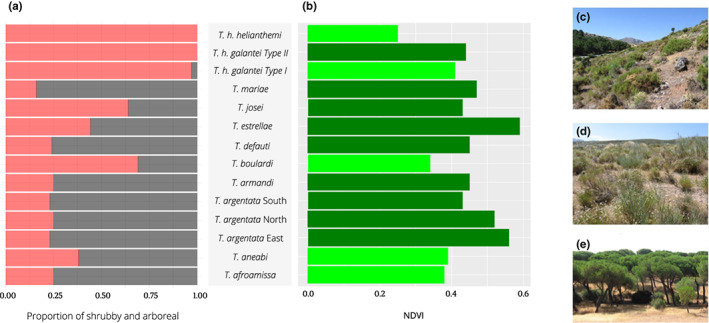
Species habitat characterization. (a) Calling site: percentage of individuals found on arboreal (grey bars) and shrubby (red bars) calling sites in each specie. (b) NDVI: average NDVI value found for each species. NDVI binary classification based on a cut‐of off 0.42 (low < 0.42 high ≥ 0.42): Dark green bars—high NDVI; light green bars—low NDVI. Showpiece of the habitat of a (c) shrubby species in a high NDVI location (*T. h. galantei* Type II in Pinos Genil, Sierra Nevada, Spain); (d) shrubby species in a low NDVI location (*T. h. helianthemi* in Caniles, Granada, Spain) and (e) arboreal species in a high NDVI location (*T. mariae* in Cartaya, Huelva, Spain).

Our final data set contained 1558 songs from 255 individuals spread across 14 species (Table 1). As expected, most species were found calling from a variety of substrates. Nine species called predominantly from trees and five from shrubs (Figure 3). *Tettigettalna. h. galantei* and *T. h. helianthemi* showed a strong preference for shrubby vegetation while *T. estrellae* was the more eclectic species.

Similarly, we found that nine species inhabit regions of high NDVI while five are found at low NDVI values. The match between the two variables is not perfect, which was anticipated, since NDVI measures vegetation greenness while the calling site measures vegetation structure. Thus, seven of the arboreal species have high NDVI values, but two arboreal species (*T. afroamissa* and *T. aneabi*) were found in regions of low NDVI, indicating that these species may purposefully choose to call from trees. In contrast, two species found in high NDVI habitats were found calling on shrubby vegetation (*T. josei* and *T. h. galantei* Type II).

### 3.2 Phylogenetic signal

Estimates of λ showed that only one acoustic variable, echeme rate, had a strong phylogenetic signal significantly different from 0 (*p* < 0.05; Table 2). The values of λ for the remaining acoustic variables and the morphological trait were not significantly different from 0 (*p* > 0.05; Table 1), indicating that these phenotypic traits are independent of shared ancestry. The estimate of D for calling site indicated a strong phylogenetic signal (*D* = −1.07; Table 2), significantly different from 1 (pRandom = 0.015), but not from the Brownian expectation of 0 (pBrownian = 0.85). This indicates that calling site preference follows a Brownian model of evolution. NDVI had a D value of 0.77 (Table 1), not significantly different from 0 (pBrownia *n* = 0.26) nor 1 (pRandom = 0.36), making it impossible to discern between a random and a Brownian model of evolution for this character. Therefore, closely related *Tettigettalna* species display similar microhabitat preferences and produce calls with similar echeme rates.

**TABLE 2:** Phylogenetic signal estimates: Maximum‐likelihood estimates of *k* and the corresponding *p*‐values for the continuous variables; Estimate of *D* and corresponding *p*‐value for the binary traits: Calling site coded as ‘arboreal’ or ‘shrubby’ and NDVI coded as ‘high’ or ‘low’.

Trait	Phylogenetic signal				
	λ	*p*	*D*	pRandom	pBrownian
log(CD)	0	1	–	–	–
log(NE)	0	1	–	–	–
log(ER)	0.978	0.040*	–	–	–
log(ED)	0.901	0.103	–	–	–
log(ID)	0.957	0.091	–	–	–
sqrt(DF)	1	0.576	–	–	–
log(PL)	0.326	0.623			
Calling site	–	–	–1.072	0.015*	0.850
NDVI	–	–	0.765	0.361	0.263

*Note*: Maximum‐likelihood estimates of MDI and the corresponding *p*‐values for the continuous variables.

Abbreviations: CD, Call duration; DF, dominant frequency; ED, echeme duration; ER, Echeme Rate; ID, Interval duration; NE, number of echemes.

* Significant results at *p* < 0.05.

### 3.3 Correlated evolution of calling song

#### 3.3.1 Body size

Phylogenetic generalized least squares analyses analysis revealed a significant negative correlation between body size (PL) and dominant frequency (PGLS: *R* ^2^ = 0.25; *p* = 0.04; Table 3; Figure S1A). There seems to be a great influence of *T. josei* in this relationship (Figure S1A). There was no association between any temporal acoustic variables and body size (Table 3). The results indicate that larger species have lower dominant frequencies, and that body size does not influence temporal patterns, which agrees with our expectations that frequencies are constrained by the size of the sound production systems.

**TABLE 3:** Phylogenetic generalized least squares model of the relationship between each acoustic variable and pronotum length (PL).

Predictor	λ	*R* ^2^ (adj)	β ± SE	*t*‐Value	*p*‐Value
log(NE)	0	−0.001			
Intercept			6.790 ± 4.039	1.681	0.119
Predictor log(PL)			−4.491 ± 4.527	−0.992	0.341
log(CD)	0	−0.071			
Intercept			2.142 ± 2.122	1.010	0.333
Predictor log(PL)			−0.876 ± 2.378	−0.368	0.719
log(ER)	0.964	−0.082			
Intercept			2.613 ± 3.747	0.697	0.499
Predictor log(PL)			−0.506 ± 4.136	−0.122	0.905
log(ID)	0.956	−0.082			
Intercept			−3.018 ± 4.474	−0.675	0.513
Predictor log(PL)			0.486 ± 4.942	0.098	0.923
log(ED)	0.947	−0.0613			
Intercept			−1.286 ± 3.540	−0.363	0.722
Predictor log(PL)			−1.953 ± 3.913	−0.499	0.627
sqrt(DF)	0.620	0.248			
Intercept			4.303 ± 0.306	14.042	8.244 × 10^−9^ *
Predictor log(PL)			−0.789 ± 0.343	−2.301	0.040*

* Significant results at *p* < 0.05.

#### 3.3.2 Climate

Nine bioclimatic variables were retained for analysis (Table 4). We found a significant relationship between two temporal acoustic variables (number of echemes and interval duration) and some of these (Table 5, Figure S1A–E). Number of echemes were negatively correlated with BIO2 (*R* ^2^ = 0.29; *p* = 0.027), BIO4 (*R* ^2^ = 0.51; *p* = 0.002) and BIO5 (*R* ^2^ = 0.26; *p* = 0.038), while interval duration was positively correlated with BIO4 (*R* ^2^ = 0.34; *p* = 0.016). These bioclimatic variables are all temperature related, BIO2 and BIO4 are measures of temperature variation and BIO5 represents maximum temperature. In climates with higher temperature variation, calling songs have a consistently lower number of echemes and longer intervals between them. Additionally, higher temperatures also trigger the production of songs with fewer echemes. These results are not in agreement with the AAH, which predicts lower frequencies in hotter and/or dryer habitats. Still, our results point to ecological selection at play. According to the *R* ^2^ values, the influence of climate was greater on the number of echemes than on interval duration and BIO4 (Temperature Seasonality) was the bioclimatic variable with the higher influence on acoustic divergence.

**TABLE 4:** Bioclimatic variables retained for analysis. Short name, long name, measurement units and definition. *Source*: O’Donnell and Ignizio (2012).

Short name	Long name	Units	Definition
BIO1	Annual Mean Temperature	Degrees Celsius	Annual average of the monthly average temperatures
BIO2	Annual Mean Diurnal Range	Degrees Celsius	Mean of the monthly temperature ranges (monthly maximum minus monthly minimum)
BIO3	Isothermality	Percent	Quantifies how large the day to‐night temperatures oscillate relative to the summer to winter (annual) oscillations
BIO4	Temperature Seasonality	Degrees Celsius	The amount of temperature variation over a given year based on the standard deviation (variation) of monthly temperature averages
BIO5	Max Temperature of Warmest Month	Degrees Celsius	The maximum monthly temperature occurrence over a given year (time‐series) or averaged span of years (normal)
BIO8	Mean Temperature of Wettest Quarter	Degrees Celsius	Quarterly index, approximates mean temperatures that prevail during the wettest season
BIO9	Mean Temperature of Driest Quarter	Degrees Celsius	Quarterly index, approximates mean temperatures that prevail during the driest quarter
BIO12	Annual Precipitation	Millimetres	Sum of all total monthly precipitation values
BIO18	Precipitation of Warmest Quarter	Millimetres	Quarterly index, approximates total precipitation that prevails during the warmest quarter

**TABLE 5:** Phylogenetic generalized least squares model of the relationship between selected (significant at *p* < 0.05) acoustic and bioclimatic variables. Results of remaining models in Table S3.

Predictor	λ	*R* ^2^ (adj)	β ± SE	*t*‐Value	*p*‐Value
log(NE)	0.000	0.290			
Intercept			8.430 ± 2.66	3.720	0.003
Predictor: bio2			−0.056 ± 0.022	−2.509	0.027
log(NE)	0.000	0.509			
Intercept			11.297 ± 2.25	5.021	0.000
Predictor: bio4			−0.002 ± 0.000	−3.803	0.003
log(NE)	0.771	0.249			
Intercept			17.677 ± 6.284	2.813	0.016
Predictor: bio5			−0.049 ± 0.021	−2.303	0.040
log(ID)	0.000	0.341			
Intercept			−10.180 ± 3.022	−3.369	0.006
Predictor: bio4			0.002 ± 0.000	2.780	0.017

#### 3.3.3 Habitat structure

**FIGURE 4:**
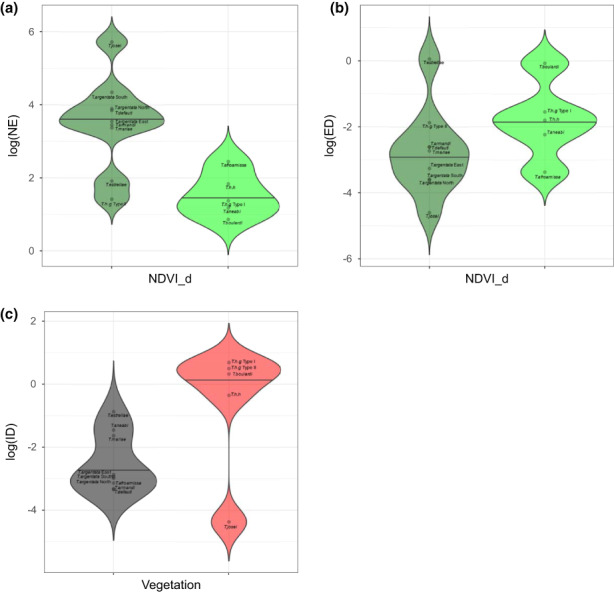
Correlated evolution between calling site and NDVI and acoustic variables. (a) NDVI and number of echemes (NE); (b) NDVI and echeme duration (ED); (c) calling site and interval duration (ID).

Phylogenetic generalized least squares analyses analysis between acoustic variables and habitat openness/closeness (NDVI, Table 6) revealed that the number of echemes (*R* ^2^ = 0.56; *p* = 0.001) and echeme duration (*R* ^2^ = 0.26; *p* = 0.036) are positively correlated with NDVI. Figure 4a,b show that species in low NDVI habitats have a lower number of echemes and longer echeme duration. The exception was *T. estrellae* and T*.h. galantei* Type II that showed a pattern similar to low NDVI species. We also found a correlation between calling site (Table 7) and interval duration (*R* ^2^ = 0.32; *p* = 0.021). Shrubby species have a higher interval duration than arboreal species (Figure 4c). The exception to this pattern is *T. josei*, with an opposite trend. Overall, species in closed habitats (high NDVI or arboreal calling site) have songs with a higher number of echemes and shorter echemes and intervals. Shorter echemes in closed habitats are predicted under the AAH, while a higher number of echemes and shorter intervals are the opposite of what is expected from the AAH.

**TABLE 6:** Phylogenetic generalized least squares model of the relationship between each acoustic variable and NDVI (high/low).

Predictor	λ	*R* ^2^ (adj)	β ± SE	*t*‐Value	*p*‐Value
log(NE)	0.851	0.559			
Intercept			4.134 ± 0.699	5.915	7.086 × 10^−05^ *
Predictor: NDVI			−2.0134 ± 0.482	−4.178	0.001*
log(CD)	0.000	0.144			
Intercept			1.604 ± 0.225	7.145	1.173 × 10^−05^ *
Predictor: NDVI			−0.670 ± 0.376	−1.784	0.100
log(ER)	0.965	0.128			
Intercept			2.524 ± 0.932	2.708	0.019
Predictor: NDVI			−0.906 ± 0.532	−1.704	0.114
log(ID)	0.939	0.1503			
Intercept			−3.036 ± 1.067	−2.846	0.015*
Predictor: NDVI			1.165 ± 0.641	1.816	0.094
log(ED)	0.978	0.260			
Intercept			−3.429 ± 0.838	−4.091	0.001*
Predictor: NDVI			1.095 ± 0.465	2.357	0.036*
sqrt(DF)	0.985	0.010			
Intercept			3.602 ± 0.101	35.735	1.474 × 10^−13*^
Predictor: NDVI			0.0857 ± 0.055	1.561	0.1445

* Significant results at *p* < 0.05.

**TABLE 7:** Phylogenetic generalized least squares model of the relationship between each acoustic variable and Calling site (shrubby/arboreal).

Predictor	λ	*R* ^2^ (adj)	β ± SE	*t*‐Value	*p*‐Value
log(NE)	0.523	0.037			
Intercept			3.654 ± 0.824	4.435	0.001*
Predictor: Calling site			−1.021 ± 0.835	−1.223	0.245
log(CD)	0	0.082			
Intercept			1.161 ± 0.233	4.992	0.000*
Predictor: Calling site			0.571 ± 0.389	1.469	0.168
log(ER)	0.931	0.168			
Intercept			2.910 ± 0.941	3.093	0.009
Predictor: Calling site			−1.517 ± 0.797	−1.904	0.081
log(ID)	0.893	0.317			
Intercept			−3.665 ± 0.981	−3.735	0.003*
Predictor: Calling site			2.238 ± 0.844	2.653	0.021*
log(ED)	0.880	−0.029			
Intercept			−3.281 ± 0.942	−3.485	0.005*
Predictor: Calling site			0.649 ± 0.814	0.798	0.441
sqrt(DF)	0.669	−0.008			
Intercept			3.580 ± 0.085	43.962	1.243 × 10^−14^
Predictor: Calling site			0.073 ± 0.077	0.946	0.363

* Significant results at *p* < 0.05.

### 3.4 Mode of acoustic evolution

**FIGURE 5:**
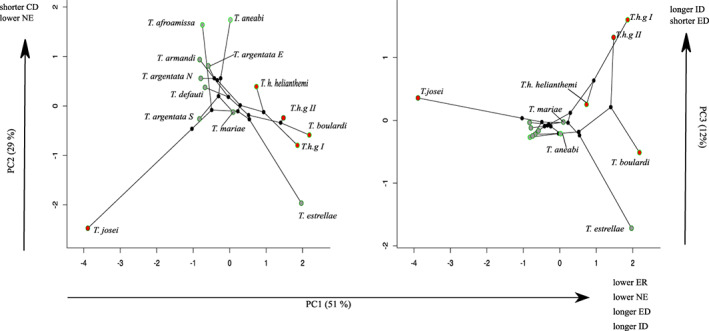
Phylomorphospace plot of temporal acoustic space. (a) PC1 vs. PC2 and (b) PC1 vs PC3. The first three principal components account for 93% of the total variation. Red dots: shrubby species; grey dots: arboreal species; dots with dark green trace: high NDVI; dots with light green trace: low NDVI. White dots: ancestral states.

A principal component analysis of acoustic temporal variables revealed that 83% of the variation can be described by the first tree axes (Figure 5). Species that score positively along PC1 (37% variation) tend to have songs with longer echemes and intervals; fewer number of echemes and lower echeme rate. Positive scores on PC 2 (28% variation) are associated with shorter calls and a lower number of echemes. While PC3 (18% variation) is linked to shorter echemes and longer intervals. Taxa distribution in the acoustic space is not phylogenetic structure as demonstrated by the crisscross of branches in the phylomorphospace (Figure 5a). A large portion of the acoustic space defined by the first two PCs is occupied by arboreal species, whereas the shrubby species have a more restricted distribution (Figure 5a). There is, however, a distinct separation between arboreal and shrubby species along PC1. This may indicate that acoustic diversification was mainly due to adaptive diversification in the environment.

Most ancestral nodes are found among the arboreal species, including the root ancestor. Notably, the ancestral node separating *T. josei* from the remaining taxa is predicted right in the middle of the acoustic space. However, there is a departure from this space by the most recent common ancestor (MRCA) between clades 1 and 2, which crossed the acoustic space to a position in between the arboreal and shrubby space. Both clades independently gave origin to a shrubby and an arboreal lineage. The two shrubby lineages proceed to differentiate further away from the space occupied by the arboreal species. Taking the opposite trajectory, the two unrelated arboreal lineages crossed the acoustic space back to arboreal space. *Tettigettalna estrellae* an arboreal species belonging to clade 1 grouped closer to the shrubby species; however, this species showed a heterogeneous calling site preference (only 56% preference for arboreal sites) being often found in low vegetation. In the space defined by PC1 and PC3 (Figure 5b) shrubby species are being further segregated along PC3, while the arboreal species are clumped together occupying a small portion of the acoustic space. Species with longer intervals and shorter echemes occupy the upper right corner, with a clear convergence between *T.h.g* I and II. *Tettigettalna boulardi* and *T. estrellae* occupy the lower right corner with seemingly parallel trajectories.

Results from the model fitting analysis (Table 8) support a Brownian Motion (BM or BMS) pattern of evolution for most temporal variables. Call duration and number of echemes diversification were best explained by Brownian Motion with strong support (ΔAICc^3^ 4). For the remaining temporal variables—echeme rate; echeme duration and interval duration—the analysis did not deliver strong support for a single model, with the three BM models (BM, BMS_Calling site_, BMS_NDVI_) conveying similar support. The spectral variable, dominant frequency diversification was best explained by an early‐burst model (EB) of evolution, although the BM model cannot be completely excluded.

**TABLE 8:** Comparison of seven evolutionary model fits, σ^*2*^: evolutionary rate parameter, global for the single‐rate models and (Calling site: shrubby/arboreal) or (NDVI: high/low) for the multi‐regime models.

Trait	Model	AIC	AICw	ΔAICc	σ^2^	Model parameters
CD	EB	42.24	0.09	4.04	0.000	*r* = −0.242
	BM	38.20	0.68	0.00	0.000	–
	OU	42.24	0.09	4.04	7.653	α = 7.780
	BMS (Calling Site)	49.59	0.00	11.39	1 × 10^−09^/0.532	θ_global_ = 1.371
	OUMV (Calling Site)	48.86	0.00	10.66	0.000/8.924	α = 6.373; θs_hrubby/arboreal_ = 1.704/1.118
	OUMV (NDVI)	42.71	0.07	4.51	11.020/0.000	α = 75.429; θ_high/low_ = 1.715/−0.134
	BMS (NDVI)	42.89	0.07	4.69	0.027/3.374	θ_global_ = 1.703
NE	EB	61.21	0.08	4.04	0.000	*r* = −2.301
	BM	57.17	0.58	0.00	0.000	–
	OU	61.21	0.08	4.04	22.413	α = 5.878
	BMS (Calling Site)	60.73	0.10	3.56	0.095/0.025	θg_lobal_ = 1.455
	OUMV (Calling Site)	66.15	0.01	8.98	0.000/2.817	α = 12.785; θ_shrubby/arboreal_ = 1.022/1.601
	OUMV (NDVI)	61.24	0.08	4.07	0.000/0.000	α = 8.365; θ_high/low_ = 1.898/1.024
	BMS (NDVI)	61.16	0.08	3.99	1 × 10^−09^/0.550	θ_global_ = 1.676
ER	EB	57.71	0.03	5.05	0.688	*r* = −0.000
	BM	53.50	0.24	0.85	0.606	–
	OU	57.54	0.03	4.89	0.649	α = 0.023
	BMS (Calling Site)	52.66	0.37	0.00	0.155/0.713	θ_global_ = 2.076
	OUMV (Calling Site)	59.88	0.01	7.22	0.131/0.631	α = 0.000; θ_shrubby/arboreal_ = −53993220/2.923
	OUMV (NDVI)	60.60	0.01	7.95	0.546/0.191	α = 0.000; θ_high/low_ = 2.455/−39099705
	BMS (NDVI)	53.04	0.31	0.38	0.591/0.263	θ_global_ = 1.962
ED	EB	56.16	0.03	5.23	0.452	*r* = −0.000
	BM	52.11	0.26	1.19	0.452	–
	OU	56.14	0.03	5.21	0.453	α = 0
	BMS (Calling Site)	52.65	0.20	1.73	0.679/0.626	θ_global_ = −3.001
	OUMV (Calling Site)	61.20	0.00	10.28	1.026/0.856	α = 0.175; θ_shrubby/arboreal_ = −1.865/−2.899
	OUMV (NDVI)	58.45	0.01	7.52	1.021/0.032	α = 0.129; θ_high/low_ = −3.361/0.669
	BMS (NDVI)	50.92	0.47	0.00	0.826/0.027	θ_global_ = −3.025
ID	EB	62.75	0.04	4.29	0.986	*r* = −0.000
	BM	58.46	0.31	0.00	0.838	–
	OU	62.46	0.04	3.99	1.069	α = 0.083
	BMS (Calling Site)	58.61	0.29	0.15	1.695/0.798	θ_global_ = −2.610
	OUMV (Calling Site)	63.82	0.02	5.36	0.283/0.959	α = 0.000; θ_shrubby/arboreal_ = 89708186/−4.212
	OUMV (NDVI)	65.24	0.01	6.77	1.147/0.496	α = 0.088; θ_high/low_ = −3.231/8.609
	BMS (NDVI)	58.64	0.29	0.18	1.065/0.631	θ_global_ = −2.599
DF	EB	−12.97	0.67	0.00	0.081	*r* = −2.448
	BM	−11.32	0.29	1.66	0.003	–
	OU	−7.27	0.04	5.70	0.003	α = 0
	BMS (Calling Site)	6.08	0.00	19.05	0.022/1.00 × 10^−09^	θ_global_ = 3.520
	OUMV (Calling Site)	12.62	0.00	25.59	1.00 × 10^−09^/1.00 × 10^−09^	α = 0.000; θ_shrubby/arboreal_ = 5898755/3.473
	OUMV (NDVI)	16.01	0.00	28.99	1.697 × 10^−03^/1.00 × 10^−09^	α = 0.000; θ_high/low_ = 3.627/−3296156
	BMS (NDVI)	7.63	0.00	20.61	0.002/1 × 10^−09^	θ_global_ = 3.587

Abbreviations: α, strength of stabilizing selection; *r*, acceleration/deceleration rate; θ, trait optimum.

## 4 DISCUSSION

Our approach in this paper was to investigate the relationship between calling song variation and ecological divergence in a phylogenetic framework while accounting for body size. We attempted to infer the evolutionary history of six acoustic parameters, and if the major forces shaping their divergence were stochastic (e.g. genetic drift) or deterministic (e.g. ecological adaptation) in nature. We included in our analysis predictions from the AAH, which postulates that acoustic communication should adapt to the physical properties of the environment in which it is produced in a way that maximizes transmission and minimizes degradation. If stochastic processes are the main driver of acoustic divergence, we expect to find no correlation between acoustic and ecological divergence and a significant phylogenetical signal; if on the other hand ecological adaptation was the most influential mechanism then acoustic divergence should relate to ecological variation and not be phylogenetically structured.

Our results indicated an interplay between stochastic and deterministic processes and different evolutionary histories for each acoustic variable. We found little support for the Acoustic Adaptation Hypothesis.

### 4.1 Phylogenetic signal and mode of evolution

We found a statistically significant phylogenetic signal in one temporal acoustic variable (echeme rate) and one ecological variable (calling site). The remaining acoustic variables, body size and the ecological variable NDVI did not display a significant phylogenetic signal. These results are in partial disagreement with the literature. Fonseca et al. (2008) contrasted the phylogenetic history of nine cicada species (including four *Tettigettalnas*) with their calling song patterns and the morphology of the song production apparatus. The authors found that the spectral traits of the song, which are strongly dependent on body size (Bennet‐Clark & Young, 1994) and the mechanisms of the tymbal (Fonseca & Popov, 1994) showed a strong correlation with phylogenetic relationships. On the contrary, the calling song temporal pattern is controlled by a highly plastic nervous system, which consequently exerts fewer biomechanical constraints on song evolution. As a result, the temporal pattern was independent of the phylogenetic history (Fonseca et al., 2008). A reasonable explanation for the lack of signal in the *spectral variable* is that our data are comprised of a single genus. Had we included other genera such as *Cicada* or *Tibicina*, both dominant frequency, and body size would probably present some degree of phylogenetic signal, as shown by Fonseca et al. (2008). Indeed, we found the expected correlation between body size and dominant frequency; around 25% of the variation in dominant frequency is explained by body size when taking phylogeny into account. Frequency ranges seem to be genus‐specific and constrained by body size (Fonseca et al., 2008), being very similar between *Tettigettalna* species, except for *T. josei*, the smallest and earliest to diverge in the genus, which calls at much higher frequencies than predicted for its size. Moreover, results from the model fitting analysis suggest an *early‐burst model* of evolution for the dominant frequency variable. It is unlikely that dominant frequency is used by female and male cicadas to distinguish conspecifics from heterospecifics of similar body size. Therefore, they probably rely on temporal parameters when found in sympatry. How females perceive these acoustic cues and whether they have an impact on mate preferences remains to be tested.

The discovery of a significant phylogenetic signal on *echeme rate* indicates that closely related species have similar values for this variable. A high phylogenetic signal is usually synonymized with evolution by neutral genetic drift. However, given that similar phylogenetic signals can be produced by several different evolutionary processes (Revell et all., 2008), results should be interpreted with caution and in conjunction with other analyses. The PGLS analysis did not reveal any correlation between echeme rate and the ecological or morphological variables. Furthermore, the model fitting analysis did not deliver strong support for a single model, but the BM and the two BMS models were similarly informative. The overall results point to evolution by *genetic drift* in this variable. The variable *call duration* had no phylogenetic signal; no correlation with any ecological or morphological variable and the model fitting analysis indicated a Brownian Motion model of evolution with strong support. Given these results, the absence of a phylogenetic signal should not in this case be interpreted as indicative of a deterministic process. Revell et all (2008), explicitly warn that a low phylogenetic signal can be produced for all but one (constant‐rate genetic drift) of the processes tested in their study. They found that a decreased phylogenetic signal can be produced under genetic drift when the rate of evolution was initially low but increased over time since this process tends to increase variation across the tips of the tree without a corresponding increase in the covariance among taxa. Consequently, the evolution of this variable seems once again best explained by *genetic drift*.

Three variables (number of echemes, echeme duration and interval duration) displayed a correlation with some ecological variables and no phylogenetic signal, suggesting ecological selection. *Interval duration* correlated with calling site in the PGLS and in the model fitting analysis, the BM and the two BMS models had similar support. Despite the lack of strong support for a single model, results from the PGLS indicate that the evolution of this variable may be connected to the calling site. The evolutionary rate parameter (σ^2^) for the BMS_(Calling Site)_ model was higher in the shrubby regime, suggesting *directional selection* in this habitat. Similarly, the variable *echeme duration* correlated with the ecological variable NDVI in the PGLS analysis. In the BMS_(NDVI)_ model, the evolutionary rate parameter (σ^2^) was higher in the high NDVI regime, which may indicate *directional selection* in habitats with high NDVI.

The variable *number of echemes* correlated with NDVI, which together with a lack of phylogenetic signal, suggests ecological selection. However, the model fitting analysis delivers strong support for the BM model in this variable. This pattern could appear under fluctuating natural selection or under strong stabilizing selection that randomly fluctuates since both these processes can be modelled by BM (Landis & Schraiber, 2017). *Fluctuating natural selection* can also produce a low phylogenetic signal when the rate of fluctuation is low (Revel et al., 2008).

### 4.2 Ecological selection and the AAH

Below, we discuss in detail the ways in which climate and vegetation may have influenced acoustic divergence, including the impact or lack thereof of the AAH.

The PGLS analysis did not retrieve the expected relationship between acoustic variables and temperature or precipitation. Overall, our results showed that species in habitats with higher temperature seasonality and higher maximum temperature have calling songs with fewer echemes and longer intervals. The mechanism through which higher annual thermic variation would lead to this song pattern is not clear. Cicada adults are active during the day and sing only in the summer months and it is unlikely that males in the same population experience very different environmental conditions during signal production. As cicada nymphs spend several years developing underground and are ectothermic, it is possible that higher temperature seasonality may affect their development differently than lower ones. Effects of developmental conditions in acoustic signals have been reported for several insects. For instance, rearing temperature modified temporal acoustic patterns of Hawaiian crickets (Grace & Shaw, 2004) and field crickets (Walker, 2000) with higher developmental temperatures resulting in faster songs. However, the sound production systems of the Orthoptera (stridulation) and the Cicadidae (by means of a tymbal mechanism) are very different (Claridge, 1985; Forrest, 1982), as well as the larval stage duration, that in cicadas can be up to 17 years underground (Williams & Simon, 1995). The most likely explanation is that the correlation found between climatic variables (BIO 2, 4 & 5) and some acoustic variables (number of echemes & interval duration) is a by‐product of the correlation between climate and vegetation. It is well documented that climatic conditions influence vegetation and plant distribution (e.g. Gavilán, 2005). Temperature seasonality (BIO4), the bioclimatic variable with greater influence on the acoustic structure, is a measure of continentality. In the Iberian Peninsula, it decreases circularly from the interior to the coasts, being particularly low on the Atlantic shores and high in the centre and south of Spain (Andaluzia) and its mountainous regions. The latter regions, particularly the high altitudes of the Sierra Nevada and Sierra de la Contraviesa are also regions of low NDVI, dominated by shrubland. A recent study using climatic data to seek the drivers of floristic composition differentiation in the thermophilous deciduous oak forests in Eastern Europe found that temperature seasonality was a major variable separating several clusters of forest types (Goncharenko et al., 2020). Indeed, we found an association between NDVI and calling song structure. Species in habitats with low NDVI (open habitat) have fewer numbers of echemes and longer echeme duration. Similarly, there is a correlation between calling site and some temporal variables. Arboreal species (closed habitat) have shorter echemes and intervals and higher echeme rates, while shrubby species (open habitat) display longer echemes and intervals and lower echeme rates. Taken together, species in open habitats have fewer number of echemes, longer echeme and interval duration, and lower echeme rate than species in closed habitats, which is the opposite pattern to the one expected under the AAH, except for echeme duration that follows expectations (shorter in closed habitats).

In the bladder cicada, *Cystosoma saunderssi*, spectral characters are more important in long‐range communication (flight), while the temporal structure is essential for short‐range communication (courtship; Doolan & Young, 1989). Similar results were found for *Tibicina haematodes* (Sueur & Aubin, 2002). Playback experiments with *C. orni* showed that interval duration may be essential for species discrimination (Simões & Quartau, 2006). The acoustic adaptation hypothesis refers especially to long‐distance signals (Ryan & Kime, 2003), since these are more susceptible to degradation. *Tettigettalna* frequencies are genus‐specific, while temporal parameters are species‐specific (Fonseca et al., 2008). It seems likely that these species use frequency for long‐distance communication and temporal proprieties for short‐distance communication, thus circumventing signal degradation. Short‐distance signals are not under strong natural selection and can even be selected for fast degradation to avoid predators and parasitoids (Endler, 1992).

In fact, is well documented that predation is an important pressure in the evolution of acoustic and visual signals. Predation of cicadas has been reported in birds (Pons, 2020; Williams et al., 1993) small mammals (Krohne et al., 1991), lizards (Han et al., 2021) and several arthropods such as spiders (Suzuki & Mukaimine, 2021) and ants (Whitford & Jackson, 2007). Cicadas are also potential hosts for phonotactic parasitoid flies. *Emblemasoma* species (Diptera: Sarcophagidae) were found parasitizing different cicada species such as *Tibicena pruinosa* and *T. chloromera* (Farris et al., 2008), *T. dorsatus* (Stucky, 2015) and *Okanagana rimosa* (Schniederkötter & Lakes‐Harlan, 2004).

A study investigating the influence of habitat structure on *E. auditrix* phonotactic strategy (Lakes‐Harlan & Kohler, 2003) found that in more structured habitats flies made more stops to reorient themselves towards their target, while in open habitats the number of direct flies was higher.

Sueur and Aubin (2003) investigating vertical segregation in two species of cicadas (*C. orni* and *T. haematodes*) in vineyards, found that segregation was not associated with signal propagation. *Cicada orni*, which displays bark mimicry, sang only lower in trunks, even though the calling song was equally propagated in both positions. On the other hand, *T. haematodes* with no cryptic coloration, called only high in the foliage, although the calling song degrades faster in this position. The authors concluded that microhabitat segregation between these two species was not linked to signal transmission, but probably to other ecological factors, such as predation avoidance.

Population density is an important factor in predation avoidance. Williams et al. (1993) found that avian predation in *Magicicada* is inversely density‐dependent due to predation satiation, reaching 100% for individuals emerging too early or too late in the season. Periodical cicadas are much easier to catch (both by humans and birds) than non‐periodic cicadas, having been labelled as ‘predatorfoolhardy’ (Lloyd & Dybas, 1966). The synchronized life cycle and large aggregations produced by these periodical cicadas are thought to have evolved precisely as an anti‐predation mechanism (Karban, 1982; Lloyd & Dybas, 1966). *Tettigettalna* species, on the other hand, have annual unsynchronized emergences and emerge at very low densities. From our observations in the field, populations in very open, shrubby habitats are particularly scarce. We also observed that males singing on shrubs are more mobile and sensitive to nearby noises, than males that sing high‐up in trees; supposedly because males calling from a tree´s canopy are better protected from predators. *Tettigettalna species* in open habitats especially those at low densities may be better off producing fast degrading signals, that attract females at short‐distance and that are harder to pinpoint by predators and parasitoids.

Signal evolution is a delicate equilibrium between local predation risk and sexual selection (Endler, 1992). Where the predation risk is higher and the strength of sexual selection lower, signals evolve to be more cryptic (Endler, 1992) or even degrade faster at shorter distances (Wiley & Richards, 1982). An extreme example is the case of the Pacific field cricket, *Teleogryllus oceanicus*, where a ‘flatwing’ mutation that causes sexual signal loss, spread in <20 generations to 5090% of the population in two Hawaiian Islands (Zuk et al., 2006, 2018). The ‘flatwing’ mutation is a rapid adaptive response to a deadly acoustically orienting parasitoid fly, whose range overlaps with *T. oceanicus* only in these islands. ‘Flatwing’ males showed increased phonotaxis to normal‐wing males and acted as ‘satellites’ to circumvent the difficulty of attracting females without song. A pre‐existing plastic behaviour coupled with strong natural selection against calling males allowed this otherwise maladaptive mutation to become established (Zuk et al., 2006)

Sexual selection has been regarded as a major driver of speciation (e.g. Fisher, 1930; Lande, 1981, 1982), this is especially so, for sexual traits, as is the case with acoustic signals (e.g. review Wilkins et al., 2013). More recently, it has been argued that speciation by (pure) sexual selection is highly unlikely and that sexual selection contributes more effectively to speciation alongside other processes, like ecological selection (Ritchie, 2007). Cornwallis and Uller (2009) argue for an ‘evolutionary ecology of sexual traits’ with a focus on how the interplay between environmental heterogeneity and phenotypic plasticity influences the evolution of sexual phenotypes.

## 5 CONCLUSION

Acoustic signals play an important role in species divergence and speciation. As such the study of their evolution is of great importance to understand the evolution of the species that rely on them for mate attraction and communication. Discerning the evolutionary process behind acoustic divergence is not always easy or even possible. By their very nature, acoustic signals are subject to a myriad of different pressures, which may act in isolation, in synergy or antagonistically to each other. Here, we present the most extensive test for acoustic ecological adaption in insects using a phylogenetic framework, including predictions of the AAH. Our results show some support for ecological adaptation at the temporal but not the spectral level, with no support for AAH.

Acoustic divergence in this group of cicadas seems to have been influenced by both stochastic and deterministic processes, acting distinctively in different variables. The most influential explanatory variables—BIO4, NDVI, and calling site—seem to reflect some measure of habitat openness. Knowing which one is the proximal variable that directly affects song structure, and which are the distal variables that act through correlation with the proximal, requires further testing. However, all three influence song structure in the same way: Songs in open habitats are defined by a low number of echemes and long echemes and intervals. These characteristics are contrary to what is expected under the AAH and can cause them to degrade more easily in these habitats. These traits are likely to have evolved as an anti‐predator strategy in conspicuous environments and low‐density populations. We hypothesize that the interplay of genetic drift (for most of the phylogenetic history) and local ecological adaptation (when faced with high‐risk habitats) played an important role in acoustic divergence in this genus.

Considering these results, it becomes relevant to verify whehter these patterns can be generalized to a higher taxonomic level. For instance, through the inclusion of related genera (e.g. *Cicadetta*) in similar analyses. Additionally, future analyses at both the intra and inter‐specific levels should include fine‐scale habitat characterization, measures of signal degradation, population density and predation risk in different environments.

While the present study emphasizes the role of ecologic selection, it does not exclude a likely role of sexual selection in the evolution of *Tettigettalna* calling songs, but it highlights the need for an integrative approach in future studies.

## AUTHOR CONTRIBUTIONS

**Raquel Mendes:** Conceptualization (lead); data curation (lead); formal analysis (lead); methodology (lead); resources (equal); software (lead); visualization (lead); writing – original draft (lead). **Vera L. Nunes:** Funding acquisition (equal); resources (equal); supervision (equal); visualization (supporting); writing – review and editing (equal). **Eduardo Marabuto:** Resources (equal); writing – review and editing (supporting). **Gonçalo J. Costa:** Resources (supporting); writing – review and editing (supporting). **Sara E. Silva:** Resources (supporting); writing – review and editing (supporting). **Octávio S. Paulo:** Supervision (equal); writing – review and editing (equal). **Paula C. Simões:** Funding acquisition (lead); supervision (lead); writing – review and editing (equal).

## CONFLICT OF INTEREST

The authors have no conflict of interest to declare.

### PEER REVIEW

The peer review history for this article is available at https://publons.com/publon/10.1111/jeb.14133.

## Supporting information


Figure S1

Table S1

Table S2

Table S3

## Data Availability

The data that support the findings of this study are openly available in Dryad at: https://doi.org/10.5061/dryad.jh9w0vtfk.
