# Supplementary material for: Testing drivers of acoustic divergence in cicadas (Cicadidae: Tettigettalna)
Source: J Evol Biol. 2022 Dec 13;36(2):461–79. doi: 10.1111/jeb.14133 (PMC10107868; doi:10.1111/jeb.14133)
Supplement: Supplementary file 2 — Table S1 [file JEB-36-461-s002.pdf]

ID	Species	Population	Country	Collector	Collection Date	Latitude	Longitude
3779	T. afroamissa	Chefchaouane (Rif mountains)	Morocco	Eduardo Marabuto	15/07/14	35,184	−5,2239
3780	T. afroamissa	Chefchaouane (Rif mountains)	Morocco	Eduardo Marabuto	15/07/14	35,184	−5,2239
3781	T. afroamissa	Chefchaouane (Rif mountains)	Morocco	Eduardo Marabuto	15/07/14	35,184	−5,2239
3782	T. afroamissa	Chefchaouane (Rif mountains)	Morocco	Eduardo Marabuto	15/07/14	35,184	−5,2239
3783	T. afroamissa	Chefchaouane (Rif mountains)	Morocco	Eduardo Marabuto	15/07/14	35,184	−5,2239
3806	T. afroamissa	East Rif	Morocco	Eduardo Marabuto	19/07/14	35,03	−4,1644
3807	T. afroamissa	East Rif	Morocco	Vera Nunes	19/07/14	35,03	−4,1644
3808	T. afroamissa	East Rif	Morocco	Vera Nunes	19/07/14	35,03	−4,1644
3813	T. afroamissa	East Rif	Morocco	Eduardo Marabuto	19/07/14	34,9652	−4,3441
3814	T. afroamissa	East Rif	Morocco	Eduardo Marabuto	19/07/14	34,9289	−4,4922
3815	T. afroamissa	East Rif	Morocco	Eduardo Marabuto	19/07/14	34,9289	−4,4922
3711	T. aneabi	Estepa, Sevilha, Andalucía	Spain	Vera Nunes	23/07/13	37,3655	−4,8183
3707	T. aneabi	Frailes, Jaen, Andalucía	Spain	Raquel Mendes	23/07/13	37,505	−3,8324
3708	T. aneabi	Frailes, Jaen, Andalucía	Spain	Raquel Mendes	23/07/13	37,505	−3,8324
3709	T. aneabi	Frailes, Jaen, Andalucía	Spain	Vera Nunes	23/07/13	37,505	−3,8324
3710	T. aneabi	Frailes, Jaen, Andalucía	Spain	Vera Nunes	23/07/13	37,505	−3,8324
3243	T. aneabi	Sierra Arana, Huetor-Santillan, Granada, Andalucía	Spain	Bruno Novais/Eduardo Marabuto/V	07-09-12	37,2557	−3,4822
3244	T. aneabi	Sierra Arana, Huetor-Santillan, Granada, Andalucía	Spain	Bruno Novais/Eduardo Marabuto/V	07-09-12	37,2557	−3,4822
3245	T. aneabi	Zagra, Granada, Andalucía	Spain	Bruno Novais/Eduardo Marabuto/V	07-10-12	37,2833	−4,2344
3246	T. aneabi	Zagra, Granada, Andalucía	Spain	Bruno Novais/Eduardo Marabuto/V	07-10-12	37,2833	−4,2344
3247	T. aneabi	Zagra, Granada, Andalucía	Spain	Bruno Novais/Eduardo Marabuto/V	07-10-12	37,2833	−4,2344
3248	T. aneabi	Zagra, Granada, Andalucía	Spain	Bruno Novais/Eduardo Marabuto/V	07-10-12	37,2833	−4,2344
3250	T. aneabi	Zagra, Granada, Andalucía	Spain	Bruno Novais/Eduardo Marabuto/V	07-10-12	37,2833	−4,2344
3252	T. aneabi	Zagra, Granada, Andalucía	Spain	Bruno Novais/Eduardo Marabuto/V	07-10-12	37,2833	−4,2344
3253	T. aneabi	Zagra, Granada, Andalucía	Spain	Bruno Novais/Eduardo Marabuto/V	07-10-12	37,2833	−4,2344
3254	T. aneabi	Zagra, Granada, Andalucía	Spain	Bruno Novais/Eduardo Marabuto/V	07-10-12	37,2833	−4,2344
3255	T. aneabi	Zagra, Granada, Andalucía	Spain	Bruno Novais/Eduardo Marabuto/V	07-10-12	37,2833	−4,2344
1	T. argentata East	Bellcaire d'Empordà, Girona, Catalunha	Spain	Pere Pons / Josep M. Bas	15/07/14	42,0688	−3,1073
2	T. argentata East	Bellcaire d'Empordà, Girona, Catalunha	Spain	Pere Pons / Josep M. Bas	15/07/14	42,0688	−3,1073
3	T. argentata East	Bellcaire d'Empordà, Girona, Catalunha	Spain	Pere Pons / Josep M. Bas	15/07/14	42,0688	−3,1073
3848	T. argentata East	Bellcaire d'Empordà, Girona, Catalunha	Spain	Pere Pons / Josep M. Bas	15/07/14	42,0688	−3,1073
3849	T. argentata East	Bellcaire d'Empordà, Girona, Catalunha	Spain	Pere Pons / Josep M. Bas	15/07/14	42,0688	−3,1073
3850	T. argentata East	Bellcaire d'Empordà, Girona, Catalunha	Spain	Pere Pons / Josep M. Bas	15/07/14	42,0688	−3,1073
3851	T. argentata East	Bellcaire d'Empordà, Girona, Catalunha	Spain	Pere Pons / Josep M. Bas	15/07/14	42,0688	−3,1073
3852	T. argentata East	Bellcaire d'Empordà, Girona, Catalunha	Spain	Pere Pons / Josep M. Bas	15/07/14	42,0688	−3,1073
3843	T. argentata East	Foixà, Girona, Catalunha	Spain	Pere Pons / Josep M. Bas	15/07/14	42,0575	2,9905
3844	T. argentata East	Foixà, Girona, Catalunha	Spain	Pere Pons / Josep M. Bas	15/07/14	42,0575	2,9905
3845	T. argentata East	Foixà, Girona, Catalunha	Spain	Pere Pons / Josep M. Bas	15/07/14	42,0575	2,9905
3846	T. argentata East	Foixà, Girona, Catalunha	Spain	Pere Pons / Josep M. Bas	15/07/14	42,0575	2,9905
3847	T. argentata East	Foixà, Girona, Catalunha	Spain	Pere Pons / Josep M. Bas	15/07/14	42,0575	2,9905
3539	T. argentata North	Albarracin, Teruel, Aragon	Spain	Eduardo Marabuto	28/06/13	40,3758	−1,3892
3547	T. argentata North	Albarracin, Teruel, Aragon	Spain	Eduardo Marabuto	28/06/13	40,4248	−1,3808
127	T. argentata North	Almaraz, Cáceres, Estremadura	Spain	Gonçalo Costa / Raquel Mendes	08-06-19	39,7709	−5,7328
131	T. argentata North	Almaraz, Cáceres, Estremadura	Spain	Gonçalo Costa / Raquel Mendes	08-06-19	39,7709	−5,7328
3525	T. argentata North	Almaraz, Cáceres, Estremadura	Spain	Eduardo Marabuto	26/06/13	39,7602	−5,7352
3526	T. argentata North	Almaraz, Cáceres, Estremadura	Spain	Eduardo Marabuto	26/06/13	39,7602	−5,7352
3528	T. argentata North	Almaraz, Cáceres, Estremadura	Spain	Eduardo Marabuto	26/06/13	39,7312	−5,7232
3529	T. argentata North	Almaraz, Cáceres, Estremadura	Spain	Eduardo Marabuto	26/06/13	39,7312	−5,7232
3838	T. argentata North	Boadella d'Empordà, Girona, Cataluna	Spain	Pere Pons / Josep M. Bas	23/07/14	42,3237	2,8586
3841	T. argentata North	Boadella d'Empordà, Girona, Cataluna	Spain	Pere Pons / Josep M. Bas	29/07/14	42,3237	2,8586
3842	T. argentata North	Boadella d'Empordà, Girona, Cataluna	Spain	Pere Pons / Josep M. Bas	29/07/14	42,3237	2,8586
3278	T. argentata North	Castelo de Vide, Portalegre	Portugal	Paula Simões	23/07/12	39,4214	−7,473
3279	T. argentata North	Castelo de Vide, Portalegre	Portugal	Paula Simões	23/07/12	39,4214	−7,473
3281	T. argentata North	Castelo de Vide, Portalegre	Portugal	Paula Simões	23/07/12	39,4214	−7,473
3283	T. argentata North	Castelo de Vide, Portalegre	Portugal	Paula Simões	23/07/12	39,3165	−7,3798
3284	T. argentata North	Castelo de Vide, Portalegre	Portugal	Paula Simões	23/07/12	39,3165	−7,3798
3285	T. argentata North	Castelo de Vide, Portalegre	Portugal	Paula Simões	23/07/12	39,3165	−7,3798
3287	T. argentata North	Castelo de Vide, Portalegre	Portugal	Paula Simões	23/07/12	39,3165	−7,3798
3288	T. argentata North	Castelo de Vide, Portalegre	Portugal	Paula Simões	23/07/12	39,3165	−7,3798
3289	T. argentata North	Castelo de Vide, Portalegre	Portugal	Paula Simões	23/07/12	39,3165	−7,3798
3290	T. argentata North	Castelo de Vide, Portalegre	Portugal	Paula Simões	23/07/12	39,3165	−7,3798
3291	T. argentata North	Castelo de Vide, Portalegre	Portugal	Paula Simões	23/07/12	39,4214	−7,473
3530	T. argentata North	Cuenca, Cuenca	Spain	Eduardo Marabuto	27/06/13	40,234	−2,0692
3853	T. argentata North	Biure d'Empordà, Girona, Cataluna	Spain	Pere Pons / Josep M. Bas	04-07-14	42,3376	2,9086
3854	T. argentata North	Biure d'Empordà, Girona, Cataluna	Spain	Pere Pons / Josep M. Bas	04-07-14	42,3376	2,9086
3274	T. argentata North	Monforte da Beira, Castelo Branco	Portugal	Paula Simões	22/07/12	39,7324	−7,3251
3275	T. argentata North	Monforte da Beira, Castelo Branco	Portugal	Paula Simões	22/07/12	39,7324	−7,3251
3276	T. argentata North	Monforte da Beira, Castelo Branco	Portugal	Paula Simões	22/07/12	39,716	−7,6282
3277	T. argentata North	Monforte da Beira, Castelo Branco	Portugal	Paula Simões	22/07/12	39,716	−7,6282
3859	T. argentata North	Montesinho, Bragança	Portugal	Genaro da Silva-Méndez	06-08-14	41,875458	−7,043158
3860	T. argentata North	Montesinho, Bragança	Portugal	Genaro da Silva-Méndez	06-08-14	41,875458	−7,043158
3025	T. argentata North	Póvoa de Lanhoso, Braga	Portugal	Bruno Novais	29/06/11	41,5818	−8,3206
3026	T. argentata North	Póvoa de Lanhoso, Braga	Portugal	Bruno Novais	29/06/11	41,5818	−8,3206

3028	T. argentata North	Póvoa de Lanhoso, Braga	Portugal	Bruno Novais	29/06/11	41,5818	−8,3206
3029	T. argentata North	Póvoa de Lanhoso, Braga	Portugal	Bruno Novais	30/06/11	41,5818	−8,3206
3037	T. argentata North	Póvoa de Lanhoso, Braga	Portugal	Bruno Novais	30/06/11	41,5818	−8,3206
3043	T. argentata North	Póvoa de Lanhoso, Braga	Portugal	Bruno Novais	01-07-11	41,5818	−8,3206
3292	T. argentata North	Serra d' Aire e Candeeiros, Santarém	Portugal	Bruno Novais	26/07/12	39,4555	−8,7998
3293	T. argentata North	Serra d' Aire e Candeeiros, Santarém	Portugal	Vera Nunes	26/07/12	39,4555	−8,7998
3294	T. argentata North	Serra d' Aire e Candeeiros, Santarém	Portugal	Vera Nunes	26/07/12	39,4555	−8,7998
3296	T. argentata North	Serra d' Aire e Candeeiros, Santarém	Portugal	Raquel Mendes	26/07/12	39,4555	−8,7998
3297	T. argentata North	Serra d' Aire e Candeeiros, Santarém	Portugal	Bruno Novais/Vera Nunes	26/07/12	39,4555	−8,7998
3299	T. argentata North	Serra d' Aire e Candeeiros, Santarém	Portugal	Bruno Novais Novais/Vera Nunes	26/07/12	39,4555	−8,7998
3300	T. argentata North	Serra d' Aire e Candeeiros, Santarém	Portugal	Bruno Novais/Vera Nunes	26/07/12	39,4555	−8,7998
3265	T. argentata North	Serra da Estrela, Covilhã, Castelo Branco	Portugal	Paula Simões	21/07/12	40,3549	−7,4402
3263	T. argentata North	Serra da Estrela, Manteigas, Guarda	Portugal	Paula Simões	21/07/12	40,3937	−7,4471
3270	T. argentata North	Serra da Estrela, Sabugal, Guarda	Portugal	Paula Simões	21/07/12	40,3103	−7,292
3271	T. argentata North	Serra da Estrela, Sabugal, Guarda	Portugal	Paula Simões	21/07/12	40,3103	−7,292
3273	T. argentata North	Serra da Estrela, Sabugal, Guarda	Portugal	Paula Simões	21/07/12	40,3103	−7,292
3840	T. argentata North	Serra de Les Avalls (Boadella d'Empordà), Girona	Spain	Pere Pons / Josep M. Bas	23/07/14	42,3237	2,8586
3839	T. argentata North	Serra de Les Avalls (Boadella d'Empordà), Girona	Spain	Pere Pons / Josep M. Bas	23/07/14	42,3237	2,8586
3000	T. argentata North	Sesimbra, Setúbal	Portugal	Bruno Novais	16/06/11	38,4469	−9,0865
3002	T. argentata North	Sesimbra, Setúbal	Portugal	Bruno Novais	16/06/11	38,4469	−9,0865
3003	T. argentata North	Sesimbra, Setúbal	Portugal	Bruno Novais	16/06/11	38,4469	−9,0865
3004	T. argentata North	Sesimbra, Setúbal	Portugal	Bruno Novais	16/06/11	38,4469	−9,0865
3005	T. argentata North	Sesimbra, Setúbal	Portugal	Bruno Novais	16/06/11	38,4434	−9,0893
3006	T. argentata North	Sesimbra, Setúbal	Portugal	Bruno Novais	16/06/11	38,4507	−9,0905
3007	T. argentata North	Sesimbra, Setúbal	Portugal	Bruno Novais	22/06/11	38,4512	−9,091
3008	T. argentata North	Sesimbra, Setúbal	Portugal	Bruno Novais	22/06/11	38,4508	−9,0907
3009	T. argentata North	Sesimbra, Setúbal	Portugal	Bruno Novais	22/06/11	38,4464	−9,0868
3010	T. argentata North	Sesimbra, Setúbal	Portugal	Bruno Novais	22/06/11	38,4429	−9,0891
3011	T. argentata North	Sesimbra, Setúbal	Portugal	Bruno Novais	22/06/11	38,4438	−9,0899
3012	T. argentata North	Sesimbra, Setúbal	Portugal	Bruno Novais	22/06/11	38,4452	−9,0909
3013	T. argentata North	Sesimbra, Setúbal	Portugal	Bruno Novais	22/06/11	38,4451	−9,0909
3014	T. argentata North	Sesimbra, Setúbal	Portugal	Bruno Novais	22/06/11	38,4426	−9,0888
3015	T. argentata North	Sesimbra, Setúbal	Portugal	Bruno Novais	22/06/11	38,4513	−9,0911
3177	T. argentata North	Sesimbra, Setúbal	Portugal	Bruno Novais/Vera Nunes	28/06/12	38,4513	−9,0911
3364	T. argentata South	Ayamonte, Huelva, Andalucía	Spain	Vera Nunes	14/08/12	37,2274	−7,3404
3118	T. argentata South	Budens, Vila do Bispo, Algarve	Portugal	Bruno Novais	27/07/11	37,0792	−8,8366
3570	T. argentata South	Cartaya, Huelva, Andalucía	Spain	Raquel Mendes	18/07/13	37,2341	−7,0638
3576	T. argentata South	Cartaya, Huelva, Andalucía	Spain	Vera Nunes	18/07/13	37,2341	−7,0638
3381	T. argentata South	Huelva, Huelva, Andalucía	Spain	Vera Nunes	16/08/12	37,1459	−6,7497
3360	T. argentata South	Mata do Lobo, Faro, Algarve	Portugal	Vera Nunes	08-08-12	37,0802	−7,9487
3161	T. argentata South	Moncarapacho, Faro, Algarve	Portugal	Bruno Novais	11-08-11	37,0782	−7,8213
3647	T. argentata South	Oria, Almeria, Andalucía	Spain	Vera Nunes	21/07/13	37,4974	−2,2921
3648	T. argentata South	Oria, Almeria, Andalucía	Spain	Vera Nunes	21/07/13	37,4974	−2,2921
3649	T. argentata South	Oria, Almeria, Andalucía	Spain	Vera Nunes	21/07/13	37,4974	−2,2921
3650	T. argentata South	Oria, Almeria, Andalucía	Spain	Vera Nunes	21/07/13	37,4974	−2,2921
3668	T. argentata South	Oria, Almeria, Andalucía	Spain	Raquel Mendes	21/07/13	37,4974	−2,2921
3669	T. argentata South	Oria, Almeria, Andalucía	Spain	Raquel Mendes	21/07/13	37,4974	−2,2921
3670	T. argentata South	Oria, Almeria, Andalucía	Spain	Raquel Mendes	21/07/13	37,4974	−2,2921
3672	T. argentata South	Oria, Almeria, Andalucía	Spain	Raquel Mendes	21/07/13	37,4974	−2,2921
3017	T. argentata South	Portel, Êvora	Portugal	Bruno Novais	28/06/11	38,3033	−7,709
3089	T. argentata South	Portel, Êvora	Portugal	Bruno Novais	21/07/11	38,3033	−7,709
3090	T. argentata South	Portel, Êvora	Portugal	Bruno Novais	21/07/11	38,3033	−7,709
3091	T. argentata South	Portel, Êvora	Portugal	Bruno Novais	21/07/11	38,3033	−7,709
3092	T. argentata South	Portel, Êvora	Portugal	Bruno Novais	21/07/11	38,3033	−7,709
3093	T. argentata South	Portel, Êvora	Portugal	Bruno Novais	21/07/11	38,3033	−7,709
3094	T. argentata South	Portel, Êvora	Portugal	Bruno Novais	21/07/11	38,3033	−7,709
3095	T. argentata South	Portel, Êvora	Portugal	Bruno Novais	21/07/11	38,3033	−7,709
3096	T. argentata South	Portel, Êvora	Portugal	Bruno Novais	21/07/11	38,3033	−7,709
3097	T. argentata South	Portel, Êvora	Portugal	Bruno Novais	21/07/11	38,3033	−7,709
3098	T. argentata South	Portel, Êvora	Portugal	Bruno Novais	21/07/11	38,3033	−7,709
3099	T. argentata South	Portel, Êvora	Portugal	Bruno Novais	21/07/11	38,3033	−7,709
3100	T. argentata South	Portel, Êvora	Portugal	Bruno Novais	21/07/11	38,3033	−7,709
3101	T. argentata South	Portel, Êvora	Portugal	Bruno Novais	21/07/11	38,3033	−7,709
3102	T. argentata South	Portel, Êvora	Portugal	Bruno Novais	21/07/11	38,3033	−7,709
3103	T. argentata South	Portel, Êvora	Portugal	Bruno Novais	21/07/11	38,3033	−7,709
3308	T. argentata South	Quinta do Lago, Faro, Algarve	Portugal	Raquel Mendes	01-08-12	37,0587	−8,0211
3324	T. argentata South	Quinta do Lago, Faro, Algarve	Portugal	Vera Nunes	02-08-12	37,0587	−8,0211
3326	T. argentata South	Quinta do Lago, Faro, Algarve	Portugal	Vera Nunes	02-08-12	37,0587	−8,0211
3328	T. argentata South	Quinta do Lago, Faro, Algarve	Portugal	Vera Nunes	02-08-12	37,0587	−8,0211
3334	T. argentata South	Quinta do Lago, Faro, Algarve	Portugal	Vera Nunes	02-08-12	37,0587	−8,0211
3344	T. argentata South	Quinta do Lago, Faro, Algarve	Portugal	Vera Nunes	03-08-12	37,0587	−8,0211
3347	T. argentata South	Quinta do Lago, Faro, Algarve	Portugal	Vera Nunes	03-08-12	37,0587	−8,0211
3356	T. argentata South	Quinta do Lago, Faro, Algarve	Portugal	Vera Nunes	08-08-12	37,0587	−8,0211
3310	T. argentata South	Quinta do Lago, Faro, Algarve	Portugal	Raquel Mendes	01-08-12	37,0587	−8,0211

3315	T. argentata South	Quinta do Lago, Faro, Algarve	Portugal	Raquel Mendes	01-08-12	37,0587	−8,0211
3321	T. argentata South	Quinta do Lago, Faro, Algarve	Portugal	Raquel Mendes	01-08-12	37,0587	−8,0211
3335	T. argentata South	Quinta do Lago, Faro, Algarve	Portugal	Raquel Mendes / Vera Nunes	02-08-12	37,0587	−8,0211
3339	T. argentata South	Quinta do Lago, Faro, Algarve	Portugal	Vera Nunes	03-08-12	37,0587	−8,0211
3348	T. argentata South	Quinta do Lago, Faro, Algarve	Portugal	Vera Nunes	07-08-12	37,0587	−8,0211
3349	T. argentata South	Quinta do Lago, Faro, Algarve	Portugal	Vera Nunes	07-08-12	37,0587	−8,0211
3357	T. argentata South	Quinta do Lago, Faro, Algarve	Portugal	Vera Nunes	08-08-12	37,0587	−8,0211
3359	T. argentata South	Quinta do Lago, Faro, Algarve	Portugal	Raquel Mendes	08-08-12	37,0587	−8,0211
3661	T. argentata South	Quinta do Lago, Faro, Algarve	Portugal	Raquel Mendes	25/07/13	37,0598	−8,0212
3730	T. argentata South	Quinta do Lago, Faro, Algarve	Portugal	Raquel Mendes	25/07/13	37,0598	−8,0212
3732	T. argentata South	Quinta do Lago, Faro, Algarve	Portugal	Raquel Mendes	25/07/13	37,0598	−8,0212
3733	T. argentata South	Quinta do Lago, Faro, Algarve	Portugal	Raquel Mendes	25/07/13	37,0598	−8,0212
3123	T. argentata South	S. Bartolomeu de Messines, Silves, Algarve	Portugal	Bruno Novais	28/07/11	37,2571	−8,2972
3124	T. argentata South	S. Bartolomeu de Messines, Silves, Algarve	Portugal	Vera	28/07/11	37,2571	−8,2972
3125	T. argentata South	S. Bartolomeu de Messines, Silves, Algarve	Portugal	Bruno Novais	28/07/11	37,2571	−8,2972
3126	T. argentata South	S. Bartolomeu de Messines, Silves, Algarve	Portugal	Bruno Novais	28/07/11	37,2571	−8,2972
3127	T. argentata South	S. Bartolomeu de Messines, Silves, Algarve	Portugal	Bruno Novais	28/07/11	37,2571	−8,2972
3128	T. argentata South	S. Bartolomeu de Messines, Silves, Algarve	Portugal	Bruno Novais	28/07/11	37,2571	−8,2972
3130	T. argentata South	S. Bartolomeu de Messines, Silves, Algarve	Portugal	Bruno Novais	28/07/11	37,2571	−8,2972
3131	T. argentata South	S. Bartolomeu de Messines, Silves, Algarve	Portugal	Bruno Novais	28/07/11	37,2571	−8,2972
3132	T. argentata South	S. Bartolomeu de Messines, Silves, Algarve	Portugal	Bruno Novais	28/07/11	37,2571	−8,2972
3133	T. argentata South	S. Bartolomeu de Messines, Silves, Algarve	Portugal	Bruno Novais	28/07/11	37,2571	−8,2972
3551	T. argentata South	S. Bartolomeu de Messines, Silves, Algarve	Portugal	Vera Nunes	17/07/13	37,2571	−8,2972
3552	T. argentata South	S. Bartolomeu de Messines, Silves, Algarve	Portugal	Vera Nunes	17/07/13	37,2571	−8,2972
3553	T. argentata South	S. Bartolomeu de Messines, Silves, Algarve	Portugal	Vera Nunes	17/07/13	37,2571	−8,2972
3146	T. argentata South	S. Brás de Alportel, Faro, Algarve	Portugal	Bruno Novais	04-08-11	37,1374	−7,8479
3979	T. argentata South	Sierra de los Filabres, Portocarrero, Almeria, Andalucía	Spain	Raquel Mendes	15/07/16	37,266	−2,5056
3980	T. argentata South	Sierra de los Filabres, Portocarrero, Almeria, Andalucía	Spain	Raquel Mendes	15/07/16	37,266	−2,5056
3256	T. argentata South	Sierra Morena, Espiel, Córdoba, Anzaluzia	Spain	Bruno Novais/Eduardo Marabuto	10-07-12	38,1844	−5,0267
3257	T. argentata South	Sierra Morena, Espiel, Córdoba, Anzaluzia	Spain	Bruno Novais/Eduardo Marabuto	10-07-12	38,1844	−5,0267
3258	T. argentata South	Sierra Morena, Espiel, Córdoba, Anzaluzia	Spain	Bruno Novais/Eduardo Marabuto	10-07-12	38,1844	−5,0267
3259	T. argentata South	Sierra Morena, Espiel, Córdoba, Anzaluzia	Spain	Bruno Novais/Eduardo Marabuto	10-07-12	38,1844	−5,0267
3260	T. argentata South	Sierra Morena, Espiel, Córdoba, Anzaluzia	Spain	Bruno Novais/Eduardo Marabuto	10-07-12	38,1844	−5,0267
3261	T. argentata South	Sierra Morena, Espiel, Córdoba, Anzaluzia	Spain	Bruno Novais/Eduardo Marabuto	10-07-12	38,1844	−5,0267
3266	T. argentata South	Sierra Morena, Espiel, Córdoba, Anzaluzia	Spain	Bruno Novais/Eduardo Marabuto	10-07-12	38,1844	−5,0267
3081	T. argentata South	Vale de Água, Santiago do Cacém	Portugal	Bruno Novais	20/07/11	37,8538	−8,6216
3082	T. argentata South	Vale de Água, Santiago do Cacém	Portugal	Bruno Novais	20/07/11	37,8538	−8,6216
3083	T. argentata South	Vale de Água, Santiago do Cacém	Portugal	Bruno Novais	20/07/11	37,8538	−8,6216
3084	T. argentata South	Vale de Água, Santiago do Cacém	Portugal	Bruno Novais	20/07/11	37,8471	−8,6182
3085	T. argentata South	Vale de Água, Santiago do Cacém	Portugal	Bruno Novais	20/07/11	37,8471	−8,6182
3086	T. argentata South	Vale de Água, Santiago do Cacém	Portugal	Bruno Novais	20/07/11	37,8531	−8,62
3087	T. argentata South	Vale de Água, Santiago do Cacém	Portugal	Bruno Novais	20/07/11	37,8531	−8,62
3088	T. argentata South	Vale de Água, Santiago do Cacém	Portugal	Bruno Novais	20/07/11	37,8531	−8,62
3104	T. argentata South	Vale de Água, Santiago do Cacém	Portugal	Bruno Novais	22/07/11	37,8531	−8,62
3173	T. argentata South	Vale do Lobo, Faro, Algarve	Portugal	Bruno Novais/Vera Nunes	27/06/12	37,0477	−8,0412
3304	T. argentata South	Vale do Lobo, Faro, Algarve	Portugal	Raquel Mendes	31/07/12	37,0639	−8,0506
3365	T. argentata South	Villablanca, Huelva, Andalucía	Spain	Vera Nunes	15/08/12	37,2676	−7,3423
3366	T. argentata South	Villablanca, Huelva, Andalucía	Spain	Vera Nunes	15/08/12	37,2676	−7,3423
3367	T. argentata South	Villablanca, Huelva, Andalucía	Spain	Vera Nunes	15/08/12	37,2676	−7,3423
3368	T. argentata South	Villablanca, Huelva, Andalucía	Spain	Vera Nunes	15/08/12	37,2676	−7,3423
3369	T. argentata South	Villablanca, Huelva, Andalucía	Spain	Raquel Mendes	15/08/12	37,2676	−7,3423
3370	T. argentata South	Villablanca, Huelva, Andalucía	Spain	Raquel Mendes	15/08/12	37,2676	−7,3423
3712	T. armandi	Estella del Marques, Cádiz, Andalucía	Spain	Vera Nunes	24/07/13	36,6846	−6,0629
3713	T. armandi	Estella del Marques, Cádiz, Andalucía	Spain	Vera Nunes	24/07/13	36,6846	−6,0629
3714	T. armandi	Estella del Marques, Cádiz, Andalucía	Spain	Vera Nunes	24/07/13	36,6846	−6,0629
3716	T. armandi	Estella del Marques, Cádiz, Andalucía	Spain	Vera Nunes	24/07/13	36,6846	−6,0629
3717	T. armandi	Estella del Marques, Cádiz, Andalucía	Spain	Vera Nunes	24/07/13	36,6846	−6,0629
3726	T. armandi	Estella del Marques, Cádiz, Andalucía	Spain	Vera Nunes	24/07/13	36,6846	−6,0629
3199	T. armandi	San Roque, Cádiz, Andalucía	Spain	Bruno Novais/Eduardo Marabuto/V	05-07-12	36,1883	−5,3593
3200	T. armandi	San Roque, Cádiz, Andalucía	Spain	Bruno Novais/Eduardo Marabuto/V	05-07-12	36,1883	−5,3593
3896	T. boulardi	Sierra de Espuña, Alhama de Murcia, Murcia	Spain	Raquel Mendes/Sara Silva	12-06-16	37,8562	−1,4949
3897	T. boulardi	Sierra de Espuña, Alhama de Murcia, Murcia	Spain	Eduardo Marabuto	12-06-16	37,8562	−1,4949
3898	T. boulardi	Sierra de Espuña, Alhama de Murcia, Murcia	Spain	Eduardo Marabuto	12-06-16	37,8562	−1,4949
3908	T. boulardi	Aguaderas, Lorca, Murcia	Spain	Eduardo Marabuto	12-06-16	37,6174	−1,5757
3909	T. boulardi	Aguaderas, Lorca, Murcia	Spain	Eduardo Marabuto	12-06-16	37,6174	−1,5757
3231	T. boulardi	Campico de los Lopez, Murcia	Spain	Bruno Novais/Eduardo Marabuto/V	08-07-12	37,5825	−1,5713
3234	T. boulardi	Campico de los Lopez, Murcia	Spain	Bruno Novais/Eduardo Marabuto/V	08-07-12	37,5825	−1,5713
4012	T. boulardi	Campo Lopez, Murcia	Spain	Raquel Mendes	07-12-17	37,5907	−1,5746
4013	T. boulardi	Campo Lopez, Murcia	Spain	Raquel Mendes	07-12-17	37,5907	−1,5746
4014	T. boulardi	Campo Lopez, Murcia	Spain	Raquel Mendes	07-12-17	37,5907	−1,5746
4015	T. boulardi	Campo Lopez, Murcia	Spain	Raquel Mendes	07-12-17	37,5907	−1,5746
4018	T. boulardi	Campo Lopez, Murcia	Spain	Raquel Mendes	07-12-17	37,5907	−1,5746
4019	T. boulardi	Campo Lopez, Murcia	Spain	Raquel Mendes	07-12-17	37,5907	−1,5746
3183	T. defauti	Puerto del Viento, Ronda, Malága	Spain	Bruno Novais/Eduardo Marabuto/V	04-07-12	36,787	−5,0533

3184	T. defauti	Puerto del Viento, Ronda, Malága	Spain	Bruno Novais/Eduardo Marabuto/V	04-07-12	36,787	−5,0533
3185	T. defauti	Puerto del Viento, Ronda, Malága	Spain	Bruno Novais/Eduardo Marabuto/V	04-07-12	36,787	−5,0533
3186	T. defauti	Puerto del Viento, Ronda, Malága	Spain	Bruno Novais/Eduardo Marabuto/V	04-07-12	36,787	−5,0533
3187	T. defauti	Puerto del Viento, Ronda, Malága	Spain	Bruno Novais/Eduardo Marabuto/V	04-07-12	36,787	−5,0533
3190	T. defauti	Serra de Grazalema, Cádiz, Andalucía	Spain	Bruno Novais/Eduardo Marabuto/V	04-07-12	36,7729	−5,2908
3216	T. defauti	Sierra Nevada, Pinos Genil, Granada, Andalucía	Spain	Bruno Novais/Eduardo Marabuto/V	07-07-12	37,1379	−3,4676
3600	T. defauti	Sierra Nevada, Pinos Genil, Granada, Andalucía	Spain	Vera Nunes	19/07/13	37,1376	−3,4761
3609	T. defauti	Sierra Nevada, Pinos Genil, Granada, Andalucía	Spain	Vera Nunes	19/07/13	37,1376	−3,4761
3611	T. defauti	Sierra Nevada, Pinos Genil, Granada, Andalucía	Spain	Vera Nunes	19/07/13	37,1376	−3,4761
3614	T. defauti	Sierra Nevada, Pinos Genil, Granada, Andalucía	Spain	Raquel Mendes	19/07/13	37,1376	−3,4761
3047	T. estrellae	Amarante, Baião, Porto	Portugal	Bruno Novais	06-07-11	41,2431	−8,0339
3049	T. estrellae	Amarante, Baião, Porto	Portugal	Bruno Novais	06-07-11	41,2436	−8,0345
3050	T. estrellae	Amarante, Baião, Porto	Portugal	Bruno Novais	06-07-11	41,2436	−8,0345
3051	T. estrellae	Amarante, Baião, Porto	Portugal	Bruno Novais	06-07-11	41,2431	−8,0339
3052	T. estrellae	Amarante, Baião, Porto	Portugal	Bruno Novais	07-07-11	41,2436	−8,0345
3019	T. estrellae	Póvoa de Lanhoso, Braga	Portugal	Bruno Novais	29/06/11	41,5818	−8,3206
3020	T. estrellae	Póvoa de Lanhoso, Braga	Portugal	Bruno Novais	29/06/11	41,5818	−8,3206
3021	T. estrellae	Póvoa de Lanhoso, Braga	Portugal	Bruno Novais	29/06/11	41,5818	−8,3206
3027	T. estrellae	Póvoa de Lanhoso, Braga	Portugal	Bruno Novais	29/06/11	41,5818	−8,3206
3030	T. estrellae	Póvoa de Lanhoso, Braga	Portugal	Bruno Novais	30/06/11	41,5818	−8,3206
3031	T. estrellae	Póvoa de Lanhoso, Braga	Portugal	Bruno Novais	30/06/11	41,5818	−8,3206
3032	T. estrellae	Póvoa de Lanhoso, Braga	Portugal	Bruno Novais	30/06/11	41,5818	−8,3206
3033	T. estrellae	Póvoa de Lanhoso, Braga	Portugal	Bruno Novais	30/06/11	41,5818	−8,3206
3035	T. estrellae	Póvoa de Lanhoso, Braga	Portugal	Bruno Novais	30/06/11	41,5818	−8,3206
3038	T. estrellae	Póvoa de Lanhoso, Braga	Portugal	Bruno Novais	30/06/11	41,5818	−8,3206
3039	T. estrellae	Póvoa de Lanhoso, Braga	Portugal	Bruno Novais	30/06/11	41,5818	−8,3206
3044	T. estrellae	Póvoa de Lanhoso, Braga	Portugal	Bruno Novais	01-07-11	41,5818	−8,3206
3045	T. estrellae	Póvoa de Lanhoso, Braga	Portugal	Bruno Novais	01-07-11	41,5818	−8,3206
3264	T. estrellae	Serra da Estrela, Covilhã, Castelo Branco	Portugal	Paula Simões	21/07/12	40,3549	−7,4402
3267	T. estrellae	Serra da Estrela, Covilhã, Castelo Branco	Portugal	Paula Simões	21/07/12	40,3549	−7,4402
3994	T. estrellae	Serra da Estrela, Sabugal, Guarda	Portugal	Raquel Mendes	03-07-17	40,3357	−7,1626
3995	T. estrellae	Serra da Estrela, Sabugal, Guarda	Portugal	Raquel Mendes	03-07-17	40,3357	−7,1626
3996	T. estrellae	Serra da Estrela, Sabugal, Guarda	Portugal	Sara Silva	03-07-17	40,3357	−7,1626
3998	T. estrellae	Serra da Estrela, Sabugal, Guarda	Portugal	Raquel Mendes	03-07-17	40,3357	−7,1626
3999	T. estrellae	Serra da Estrela, Sabugal, Guarda	Portugal	Raquel Mendes	03-07-17	40,3357	−7,1626
4000	T. estrellae	Serra da Estrela, Sabugal, Guarda	Portugal	Raquel Mendes	03-07-17	40,3357	−7,1626
3272	T. estrellae	Serra da Estrela, Sabugal, Guarda	Portugal	Paula Simões	21/07/12	40,3103	−7,292
3562	T. josei	Cartaya, Huelva, Andalucía	Spain	Vera Nunes	18/07/13	37,2607	−7,1288
3566	T. josei	Cartaya, Huelva, Andalucía	Spain	Vera Nunes	18/07/13	37,2607	−7,1288
3135	T. josei	Castro Marim, Faro, Algarve	Portugal	Bruno Novais	02-08-11	37,1864	−7,4839
3138	T. josei	Castro Marim, Faro, Algarve	Portugal	Bruno Novais	02-08-11	37,1864	−7,4839
3105	T. josei	Lagoa, Faro, Algarve	Portugal	Bruno Novais	26/07/11	37,1359	−8,3845
3110	T. josei	Lagoa, Faro, Algarve	Portugal	Bruno Novais	26/07/11	37,1359	−8,3845
3114	T. josei	Lagoa, Faro, Algarve	Portugal	Bruno Novais	27/07/11	37,1361	−8,386
3309	T. josei	Quinta do Lago, Faro, Algarve	Portugal	Vera Nunes	01-08-12	37,0587	−8,0211
3311	T. josei	Quinta do Lago, Faro, Algarve	Portugal	Vera Nunes	01-08-12	37,0587	−8,0211
3312	T. josei	Quinta do Lago, Faro, Algarve	Portugal	Vera Nunes	01-08-12	37,0587	−8,0211
3314	T. josei	Quinta do Lago, Faro, Algarve	Portugal	Vera Nunes	01-08-12	37,0587	−8,0211
3316	T. josei	Quinta do Lago, Faro, Algarve	Portugal	Vera Nunes	01-08-12	37,0587	−8,0211
3318	T. josei	Quinta do Lago, Faro, Algarve	Portugal	Vera Nunes	01-08-12	37,0587	−8,0211
3331	T. josei	Quinta do Lago, Faro, Algarve	Portugal	Vera Nunes	01-08-12	37,0587	−8,0211
3332	T. josei	Quinta do Lago, Faro, Algarve	Portugal	Vera Nunes	01-08-12	37,0587	−8,0211
3333	T. josei	Quinta do Lago, Faro, Algarve	Portugal	Vera Nunes	01-08-12	37,0587	−8,0211
3353	T. josei	Quinta do Lago, Faro, Algarve	Portugal	Vera Nunes	01-08-12	37,0587	−8,0211
3355	T. josei	Quinta do Lago, Faro, Algarve	Portugal	Vera Nunes	01-08-12	37,0587	−8,0211
3362	T. josei	Quinta do Lago, Faro, Algarve	Portugal	Vera Nunes	01-08-12	37,0587	−8,0211
3145	T. josei	S. Brás de Alportel, Faro, Algarve	Portugal	Bruno Novais	04-08-11	37,1374	−7,8479
3056	T. josei	Vale Judeu, Faro, Algarve	Portugal	Bruno Novais	12-07-11	37,1277	−8,0934
3057	T. josei	Vale Judeu, Faro, Algarve	Portugal	Bruno Novais	12-07-11	37,1277	−8,0934
3060	T. josei	Vale Judeu, Faro, Algarve	Portugal	Bruno Novais	12-07-11	37,1277	−8,0934
3061	T. josei	Vale Judeu, Faro, Algarve	Portugal	Bruno Novais	12-07-11	37,1277	−8,0934
3062	T. josei	Vale Judeu, Faro, Algarve	Portugal	Bruno Novais	12-07-11	37,1277	−8,0934
3063	T. josei	Vale Judeu, Faro, Algarve	Portugal	Bruno Novais	12-07-11	37,1277	−8,0934
3064	T. josei	Vale Judeu, Faro, Algarve	Portugal	Bruno Novais	12-07-11	37,1277	−8,0934
3373	T. mariae	Cartaya, Huelva, Andalucía	Spain	Raquel Mendes / Vera Nunes	15/08/12	37,2623	−7,1303
3374	T. mariae	Cartaya, Huelva, Andalucía	Spain	Raquel Mendes / Vera Nunes	15/08/12	37,2623	−7,1303
3375	T. mariae	Cartaya, Huelva, Andalucía	Spain	Raquel Mendes / Vera Nunes	15/08/12	37,2623	−7,1303
3376	T. mariae	Cartaya, Huelva, Andalucía	Spain	Raquel Mendes / Vera Nunes	15/08/12	37,2623	−7,1303
3378	T. mariae	Cartaya, Huelva, Andalucía	Spain	Vera Nunes	15/08/12	37,2623	−7,1303
3556	T. mariae	Cartaya, Huelva, Andalucía	Spain	Vera Nunes	17/07/13	37,2607	−7,1288
3558	T. mariae	Cartaya, Huelva, Andalucía	Spain	Vera Nunes	17/07/13	37,2607	−7,1288
3560	T. mariae	Cartaya, Huelva, Andalucía	Spain	Vera Nunes	18/07/13	37,2256	−7,0348
3561	T. mariae	Cartaya, Huelva, Andalucía	Spain	Vera Nunes	18/07/13	37,2256	−7,0348
3563	T. mariae	Cartaya, Huelva, Andalucía	Spain	Raquel Mendes	18/07/13	37,2607	−7,1288

3564	T. mariae	Cartaya, Huelva, Andalucía	Spain	Raquel Mendes	18/07/13	37,2607	−7,1288
3565	T. mariae	Cartaya, Huelva, Andalucía	Spain	Raquel Mendes	18/07/13	37,2607	−7,1288
3568	T. mariae	Cartaya, Huelva, Andalucía	Spain	Raquel Mendes	18/07/13	37,2341	−7,0638
3569	T. mariae	Cartaya, Huelva, Andalucía	Spain	Raquel Mendes	18/07/13	37,2341	−7,0638
3718	T. mariae	Cartaya, Huelva, Andalucía	Spain	Vera Nunes	24/07/13	37,2256	−7,0348
3720	T. mariae	Cartaya, Huelva, Andalucía	Spain	Vera Nunes	24/07/13	37,2256	−7,0348
3721	T. mariae	Cartaya, Huelva, Andalucía	Spain	Vera Nunes	24/07/13	37,2256	−7,0348
3722	T. mariae	Cartaya, Huelva, Andalucía	Spain	Vera Nunes	24/07/13	37,2256	−7,0348
3174	T. mariae	Corgo da Zorra, Faro, Algarve	Portugal	Bruno Novais/Vera Nunes	27/06/12	37,0556	−8,0406
3176	T. mariae	Corgo da Zorra, Faro, Algarve	Portugal	Bruno Novais/Vera Nunes	28/06/12	37,0613	−8,0383
3302	T. mariae	Corgo da Zorra, Faro, Algarve	Portugal	Raquel Mendes	31/07/12	37,0726	−8,0493
3303	T. mariae	Corgo da Zorra, Faro, Algarve	Portugal	Raquel Mendes	31/07/12	37,0726	−8,0493
3380	T. mariae	Huelva, Huelva, Andalucía	Spain	Raquel Mendes / Vera Nunes	16/08/12	37,2085	−6,7795
3382	T. mariae	Huelva, Huelva, Andalucía	Spain	Vera Nunes	16/08/12	37,2119	−6,5642
3727	T. mariae	Quinta do Lago, Faro, Algarve	Portugal	Vera Nunes	25/07/13	37,0598	−8,0212
3305	T. mariae	Quinta do Lago, Faro, Algarve	Portugal	Raquel Mendes	01-08-12	37,0587	−8,0211
3313	T. mariae	Quinta do Lago, Faro, Algarve	Portugal	Vera Nunes	01-08-12	37,0587	−8,0211
3317	T. mariae	Quinta do Lago, Faro, Algarve	Portugal	Vera Nunes	01-08-12	37,0587	−8,0211
3325	T. mariae	Quinta do Lago, Faro, Algarve	Portugal	Vera Nunes	02-08-12	37,0587	−8,0211
3327	T. mariae	Quinta do Lago, Faro, Algarve	Portugal	Vera Nunes	02-08-12	37,0587	−8,0211
3329	T. mariae	Quinta do Lago, Faro, Algarve	Portugal	Raquel Mendes	02-08-12	37,0587	−8,0211
3330	T. mariae	Quinta do Lago, Faro, Algarve	Portugal	Vera Nunes	02-08-12	37,0587	−8,0211
3336	T. mariae	Quinta do Lago, Faro, Algarve	Portugal	Raquel Mendes / Vera Nunes	02-08-12	37,0587	−8,0211
3337	T. mariae	Quinta do Lago, Faro, Algarve	Portugal	Vera Nunes	02-08-12	37,0587	−8,0211
3340	T. mariae	Quinta do Lago, Faro, Algarve	Portugal	Raquel Mendes	03-08-12	37,0587	−8,0211
3341	T. mariae	Quinta do Lago, Faro, Algarve	Portugal	Vera Nunes	03-08-12	37,0587	−8,0211
3342	T. mariae	Quinta do Lago, Faro, Algarve	Portugal	Raquel Mendes	03-08-12	37,0587	−8,0211
3343	T. mariae	Quinta do Lago, Faro, Algarve	Portugal	Raquel Mendes	03-08-12	37,0587	−8,0211
3352	T. mariae	Quinta do Lago, Faro, Algarve	Portugal	Vera Nunes	08-08-12	37,0587	−8,0211
3354	T. mariae	Quinta do Lago, Faro, Algarve	Portugal	Vera Nunes	08-08-12	37,0587	−8,0211
3363	T. mariae	Quinta do Lago, Faro, Algarve	Portugal	Raquel Mendes	09-08-12	37,0587	−8,0211
3143	T. mariae	Vale do Lobo, Faro, Algarve	Portugal	Bruno Novais	04-08-11	37,0614	−8,0609
3144	T. mariae	Vale do Lobo, Faro, Algarve	Portugal	Bruno Novais	04-08-11	37,0614	−8,0609
3147	T. mariae	Vale do Lobo, Faro, Algarve	Portugal	Bruno Novais	05-08-11	37,0614	−8,0609
3148	T. mariae	Vale do Lobo, Faro, Algarve	Portugal	Bruno Novais	05-08-11	37,0614	−8,0609
3151	T. mariae	Vale do Lobo, Faro, Algarve	Portugal	Bruno Novais	10-08-11	37,0614	−8,0609
3152	T. mariae	Vale do Lobo, Faro, Algarve	Portugal	Bruno Novais	10-08-11	37,0614	−8,0609
3153	T. mariae	Vale do Lobo, Faro, Algarve	Portugal	Bruno Novais	10-08-11	37,0614	−8,0609
3155	T. mariae	Vale do Lobo, Faro, Algarve	Portugal	Bruno Novais	11-08-11	37,0614	−8,0609
3157	T. mariae	Vale do Lobo, Faro, Algarve	Portugal	Bruno Novais	11-08-11	37,0614	−8,0609
3158	T. mariae	Vale do Lobo, Faro, Algarve	Portugal	Bruno Novais	11-08-11	37,0614	−8,0609
3166	T. mariae	Vale Judeu, Faro, Algarve	Portugal	Bruno Novais/Vera Nunes	26/06/12	37,1059	−8,0952
3168	T. mariae	Vale Judeu, Faro, Algarve	Portugal	Bruno Novais/Vera Nunes	26/06/12	37,112	−8,2025
3169	T. mariae	Vale Judeu, Faro, Algarve	Portugal	Bruno Novais/Vera Nunes	26/06/12	37,1171	−8,2094
3067	T. mariae	Vale Judeu, Faro, Algarve	Portugal	Bruno Novais	14/07/11	37,1058	−8,0952
3068	T. mariae	Vale Judeu, Faro, Algarve	Portugal	Bruno Novais	14/07/11	37,1058	−8,0952
3069	T. mariae	Vale Judeu, Faro, Algarve	Portugal	Bruno Novais	14/07/11	37,1058	−8,0952
3070	T. mariae	Vale Judeu, Faro, Algarve	Portugal	Bruno Novais	14/07/11	37,1058	−8,0952
3071	T. mariae	Vale Judeu, Faro, Algarve	Portugal	Bruno Novais	14/07/11	37,1058	−8,0952
3072	T. mariae	Vale Judeu, Faro, Algarve	Portugal	Bruno Novais	14/07/11	37,1058	−8,0952
3073	T. mariae	Vale Judeu, Faro, Algarve	Portugal	Bruno Novais	14/07/11	37,1058	−8,0952
3074	T. mariae	Vale Judeu, Faro, Algarve	Portugal	Bruno Novais	14/07/11	37,1058	−8,0952
3075	T. mariae	Vale Judeu, Faro, Algarve	Portugal	Bruno Novais	14/07/11	37,1058	−8,0952
3076	T. mariae	Vale Judeu, Faro, Algarve	Portugal	Bruno Novais	14/07/11	37,1058	−8,0952
3077	T. mariae	Vale Judeu, Faro, Algarve	Portugal	Bruno Novais	14/07/11	37,1058	−8,0952
3078	T. mariae	Vale Judeu, Faro, Algarve	Portugal	Bruno Novais	14/07/11	37,1058	−8,0952
3079	T. mariae	Vale Judeu, Faro, Algarve	Portugal	Bruno Novais	14/07/11	37,1058	−8,0952
3080	T. mariae	Vale Judeu, Faro, Algarve	Portugal	Bruno Novais	14/07/11	37,1058	−8,0952
3729	T. mariae	Vale Judeu, Faro, Algarve	Portugal	Vera Nunes	25/07/13	37,1058	−8,0952
3170	T. mariae	Vale Judeu, Faro, Algarve	Portugal	Bruno Novais/Vera Nunes	27/06/12	37,0686	−8,0296
3206	T.h. galantei Type I	Capileira, Granada, Andalucía	Spain	Bruno Novais/Eduardo Marabuto/V	06-07-12	36,9567	−3,3531
3207	T.h. galantei Type I	Capileira, Granada, Andalucía	Spain	Bruno Novais/Eduardo Marabuto/V	06-07-12	36,9567	−3,3531
3209	T.h. galantei Type I	Capileira, Granada, Andalucía	Spain	Bruno Novais/Eduardo Marabuto/V	06-07-12	36,9565	−3,3474
3211	T.h. galantei Type I	Capileira, Granada, Andalucía	Spain	Bruno Novais/Eduardo Marabuto/V	06-07-12	36,9565	−3,3474
3212	T.h. galantei Type I	Capileira, Granada, Andalucía	Spain	Bruno Novais/Eduardo Marabuto/V	06-07-12	36,9565	−3,3474
3213	T.h. galantei Type I	Capileira, Granada, Andalucía	Spain	Bruno Novais/Eduardo Marabuto/V	06-07-12	36,9565	−3,3474
3214	T.h. galantei Type I	Capileira, Granada, Andalucía	Spain	Bruno Novais/Eduardo Marabuto/V	06-07-12	36,9565	−3,3474
3676	T.h. galantei Type I	Sierra de la Contraviesa, Granada, Andalucía	Spain	Raquel Mendes	22/07/13	36,8714	−3,2267
3677	T.h. galantei Type I	Sierra de la Contraviesa, Granada, Andalucía	Spain	Raquel Mendes	22/07/13	36,8714	−3,2267
3679	T.h. galantei Type I	Sierra de la Contraviesa, Granada, Andalucía	Spain	Raquel Mendes	22/07/13	36,8714	−3,2267
3682	T.h. galantei Type I	Sierra de la Contraviesa, Granada, Andalucía	Spain	Vera Nunes	22/07/13	36,8714	−3,2267
3683	T.h. galantei Type I	Sierra de la Contraviesa, Granada, Andalucía	Spain	Vera Nunes	22/07/13	36,8714	−3,2267
3684	T.h. galantei Type I	Sierra de la Contraviesa, Granada, Andalucía	Spain	Vera Nunes	22/07/13	36,8714	−3,2267
3685	T.h. galantei Type I	Sierra de la Contraviesa, Granada, Andalucía	Spain	Vera Nunes	22/07/13	36,8714	−3,2267

3697	T.h. galantei Type I	Sierra de la Contraviesa, Granada, Andalucía	Spain	Vera Nunes	22/07/13	36,8714	−3,2267
3698	T.h. galantei Type I	Sierra de la Contraviesa, Granada, Andalucía	Spain	Vera Nunes	22/07/13	36,8714	−3,2267
3699	T.h. galantei Type I	Sierra de la Contraviesa, Granada, Andalucía	Spain	Vera Nunes	22/07/13	36,8714	−3,2267
3700	T.h. galantei Type I	Sierra de la Contraviesa, Granada, Andalucía	Spain	Vera Nunes	22/07/13	36,8714	−3,2267
3239	T.h. galantei Type I	Sierra Nevada, Laroles , Granada, Andalucía	Spain	Bruno Novais/Eduardo Marabuto/V	09-07-12	37,0493	−3,0169
3242	T.h. galantei Type I	Sierra Nevada, Laroles , Granada, Andalucía	Spain	Bruno Novais/Eduardo Marabuto/V	09-07-12	37,0493	−3,0169
3673	T.h. galantei Type I	Sierra Nevada, Narila , Granada, Andalucía	Spain	Raquel Mendes	22/07/13	36,9601	−3,1753
3674	T.h. galantei Type I	Sierra Nevada, Narila , Granada, Andalucía	Spain	Raquel Mendes	22/07/13	36,9601	−3,1753
3675	T.h. galantei Type I	Sierra Nevada, Narila , Granada, Andalucía	Spain	Raquel Mendes	22/07/13	36,9601	−3,1753
3691	T.h. galantei Type I	Sierra Nevada, Narila , Granada, Andalucía	Spain	Vera Nunes	22/07/13	36,9601	−3,1753
3692	T.h. galantei Type I	Sierra Nevada, Narila , Granada, Andalucía	Spain	Vera Nunes	22/07/13	36,9601	−3,1753
3694	T.h. galantei Type I	Sierra Nevada, Narila , Granada, Andalucía	Spain	Raquel Mendes	22/07/13	36,9601	−3,1753
3695	T.h. galantei Type I	Sierra Nevada, Narila , Granada, Andalucía	Spain	Raquel Mendes	22/07/13	36,9601	−3,1753
3696	T.h. galantei Type I	Sierra Nevada, Narila , Granada, Andalucía	Spain	Raquel Mendes	22/07/13	36,9601	−3,1753
3687	T.h. galantei Type I	Sierra Nevada, Rubite, Granada, Andalucía	Spain	Raquel Mendes	22/07/13	36,8217	−3,3347
3688	T.h. galantei Type I	Sierra Nevada, Rubite, Granada, Andalucía	Spain	Raquel Mendes	22/07/13	36,8217	−3,3347
3689	T.h. galantei Type I	Sierra Nevada, Rubite, Granada, Andalucía	Spain	Raquel Mendes	22/07/13	36,8217	−3,3347
3704	T.h. galantei Type I	Sierra Nevada, Rubite, Granada, Andalucía	Spain	Vera Nunes	22/07/13	36,8217	−3,3347
3705	T.h. galantei Type I	Sierra Nevada, Rubite, Granada, Andalucía	Spain	Vera Nunes	22/07/13	36,8217	−3,3347
3706	T.h. galantei Type I	Sierra Nevada, Rubite, Granada, Andalucía	Spain	Vera Nunes	22/07/13	36,8217	−3,3347
3578	T.h. galantei Type II	Sierra Nevada, Lanjaron, Granada, Andalucía	Spain	Raquel Mendes	19/07/13	36,9227	−3,5314
3580	T.h. galantei Type II	Sierra Nevada, Lanjaron, Granada, Andalucía	Spain	Raquel Mendes	19/07/13	36,9227	−3,5314
3585	T.h. galantei Type II	Sierra Nevada, Lanjaron, Granada, Andalucía	Spain	Raquel Mendes	19/07/13	36,9227	−3,5314
3586	T.h. galantei Type II	Sierra Nevada, Lanjaron, Granada, Andalucía	Spain	Raquel Mendes	19/07/13	36,9227	−3,5314
3589	T.h. galantei Type II	Sierra Nevada, Lanjaron, Granada, Andalucía	Spain	Vera Nunes	19/07/13	36,9227	−3,5314
3590	T.h. galantei Type II	Sierra Nevada, Lanjaron, Granada, Andalucía	Spain	Vera Nunes	19/07/13	36,9227	−3,5314
3591	T.h. galantei Type II	Sierra Nevada, Lanjaron, Granada, Andalucía	Spain	Vera Nunes	19/07/13	36,9227	−3,5314
3594	T.h. galantei Type II	Sierra Nevada, Lanjaron, Granada, Andalucía	Spain	Vera Nunes	19/07/13	36,9227	−3,5314
3201	T.h. galantei Type II	Sierra Nevada, Lanjaron, Granada, Andalucía	Spain	Bruno Novais/Eduardo Marabuto/V	06-07-12	36,9227	−3,5314
3204	T.h. galantei Type II	Sierra Nevada, Lanjaron, Granada, Andalucía	Spain	Bruno Novais/Eduardo Marabuto/V	06-07-12	36,9161	−3,504
3205	T.h. galantei Type II	Sierra Nevada, Lanjaron, Granada, Andalucía	Spain	Bruno Novais/Eduardo Marabuto/V	06-07-12	36,9161	−3,504
3615	T.h. galantei Type II	Sierra Nevada, Pinos Genil, Granada, Andalucía	Spain	Vera Nunes	20/07/13	37,1376	−3,4761
3617	T.h. galantei Type II	Sierra Nevada, Pinos Genil, Granada, Andalucía	Spain	Raquel Mendes	20/07/13	37,1376	−3,4761
3618	T.h. galantei Type II	Sierra Nevada, Pinos Genil, Granada, Andalucía	Spain	Raquel Mendes	20/07/13	37,1376	−3,4761
3619	T.h. galantei Type II	Sierra Nevada, Pinos Genil, Granada, Andalucía	Spain	Vera Nunes	20/07/13	37,1376	−3,4761
3622	T.h. galantei Type II	Sierra Nevada, Pinos Genil, Granada, Andalucía	Spain	Vera Nunes	20/07/13	37,1376	−3,4761
3623	T.h. galantei Type II	Sierra Nevada, Pinos Genil, Granada, Andalucía	Spain	Vera Nunes	20/07/13	37,1376	−3,4761
3232	T.h. galantei Type II	Sierra Nevada, Pinos Genil, Granada, Andalucía	Spain	Bruno Novais/Eduardo Marabuto/V	07-07-12	37,1379	−3,4676
3219	T.h. helianthemi	Cabo da Gata, Almeria, Andalucía	Spain	Bruno Novais/Eduardo Marabuto/V	08-07-12	36,8384	−2,2932
3220	T.h. helianthemi	Cabo da Gata, Almeria, Andalucía	Spain	Bruno Novais/Eduardo Marabuto/V	08-07-12	36,8384	−2,2932
3221	T.h. helianthemi	Cabo da Gata, Almeria, Andalucía	Spain	Bruno Novais/Eduardo Marabuto/V	08-07-12	36,8384	−2,2932
3222	T.h. helianthemi	Cabo da Gata, Almeria, Andalucía	Spain	Bruno Novais/Eduardo Marabuto/V	08-07-12	36,8384	−2,2932
3224	T.h. helianthemi	Cabo da Gata, Almeria, Andalucía	Spain	Bruno Novais/Eduardo Marabuto/V	08-07-12	36,8384	−2,2932
3225	T.h. helianthemi	Cabo da Gata, Almeria, Andalucía	Spain	Bruno Novais/Eduardo Marabuto/V	08-07-12	36,8384	−2,2932
3226	T.h. helianthemi	Cabo da Gata, Almeria, Andalucía	Spain	Bruno Novais/Eduardo Marabuto/V	08-07-12	36,8384	−2,2932
3227	T.h. helianthemi	Cabo da Gata, Almeria, Andalucía	Spain	Bruno Novais/Eduardo Marabuto/V	08-07-12	36,8384	−2,2932
3228	T.h. helianthemi	Cabo da Gata, Almeria, Andalucía	Spain	Bruno Novais/Eduardo Marabuto/V	08-07-12	36,8384	−2,2932
3230	T.h. helianthemi	Cabo da Gata, Almeria, Andalucía	Spain	Bruno Novais/Eduardo Marabuto/V	08-07-12	36,8384	−2,2932
3641	T.h. helianthemi	Cantoria, Almeria, Andalucía	Spain	Vera Nunes	21/07/13	37,3446	−2,199
3642	T.h. helianthemi	Cantoria, Almeria, Andalucía	Spain	Raquel Mendes	21/07/13	37,3446	−2,199
3644	T.h. helianthemi	Cantoria, Almeria, Andalucía	Spain	Vera Nunes	21/07/13	37,3446	−2,199
3645	T.h. helianthemi	Cantoria, Almeria, Andalucía	Spain	Vera Nunes	21/07/13	37,3446	−2,199
3664	T.h. helianthemi	Cantoria, Almeria, Andalucía	Spain	Raquel Mendes	21/07/13	37,3446	−2,199
3665	T.h. helianthemi	Cantoria, Almeria, Andalucía	Spain	Raquel Mendes	21/07/13	37,3446	−2,199
3666	T.h. helianthemi	Cantoria, Almeria, Andalucía	Spain	Raquel Mendes	21/07/13	37,3446	−2,199
3667	T.h. helianthemi	Cantoria, Almeria, Andalucía	Spain	Raquel Mendes	21/07/13	37,3446	−2,199
3624	T.h. helianthemi	PNSBaza, Caniles, Granada, Andalucía	Spain	Vera Nunes	20/07/13	37,3663	−2,7324
3626	T.h. helianthemi	PNSBaza, Caniles, Granada, Andalucía	Spain	Vera Nunes	20/07/13	37,3663	−2,7324
3628	T.h. helianthemi	PNSBaza, Caniles, Granada, Andalucía	Spain	Vera Nunes	20/07/13	37,3663	−2,7324
3629	T.h. helianthemi	PNSBaza, Caniles, Granada, Andalucía	Spain	Vera Nunes	20/07/13	37,3663	−2,7324
3630	T.h. helianthemi	PNSBaza, Caniles, Granada, Andalucía	Spain	Vera Nunes	20/07/13	37,3663	−2,7324
3631	T.h. helianthemi	PNSBaza, Caniles, Granada, Andalucía	Spain	Vera Nunes	20/07/13	37,3663	−2,7324
3937	T.h. helianthemi	PNSBaza, Caniles, Granada, Andalucía	Spain	Raquel Mendes	14/07/16	37,4297	−2,7158
3938	T.h. helianthemi	PNSBaza, Caniles, Granada, Andalucía	Spain	Raquel Mendes	14/07/16	37,4297	−2,7158
3951	T.h. helianthemi	PNSBaza, Caniles, Granada, Andalucía	Spain	Raquel Mendes	14/07/16	37,3664	−2,7331
3954	T.h. helianthemi	PNSBaza, Caniles, Granada, Andalucía	Spain	Raquel Mendes	14/07/16	37,3664	−2,7331
3956	T.h. helianthemi	PNSBaza, Caniles, Granada, Andalucía	Spain	Raquel Mendes	14/07/16	37,3664	−2,7331
3957	T.h. helianthemi	PNSBaza, Caniles, Granada, Andalucía	Spain	Raquel Mendes	14/07/16	37,3664	−2,7331
3958	T.h. helianthemi	PNSBaza, Caniles, Granada, Andalucía	Spain	Raquel Mendes	14/07/16	37,3664	−2,7331
3959	T.h. helianthemi	PNSBaza, Caniles, Granada, Andalucía	Spain	Raquel Mendes	14/07/16	37,3664	−2,7331
3962	T.h. helianthemi	PNSBaza, Caniles, Granada, Andalucía	Spain	Raquel Mendes	14/07/16	37,3391	−2,7603
3895	T.h. helianthemi	Pulpite, Cúllar, Granada, Andalucía	Spain	Eduardo Marabuto	11-06-16	37,5764	−2,5077
3236	T.h. helianthemi	Vera, Almeria, Andalucía	Spain	Bruno Novais/Eduardo Marabuto/V	08-07-12	37,2133	−1,8996
3237	T.h. helianthemi	Vera, Almeria, Andalucía	Spain	Bruno Novais/Eduardo Marabuto/V	08-07-12	37,2133	−1,8996

3238	T.h. helianthemi	Vera, Almeria, Andalucía	Spain	Bruno Novais/Eduardo Marabuto/V 08-07-12	37,2133	−1,8996
