# Supplementary material for: Testing drivers of acoustic divergence in cicadas (Cicadidae: Tettigettalna)
Source: J Evol Biol. 2022 Dec 13;36(2):461–79. doi: 10.1111/jeb.14133 (PMC10107868; doi:10.1111/jeb.14133)
Supplement: Supplementary file 3 — Table S2 [file JEB-36-461-s004.pdf]

SP	CD	NE	ER	ED	ID	DF
TAFR	0.85957	11.55583	13.69834	0.03439	0.04344	11.88878
TANE	0.85755	3.24172	3.90236	0.10705	0.23447	12.9704
TARG_North	3.70978	49.35557	13.15477	0.02774	0.05077	11.47853
TARG_South	5.86769	77.2994	12.92789	0.02705	0.05344	12.09283
TARG_Easth	3.27962	34.72222	10.78048	0.03846	0.05688	12.91467
TBOU	4.43516	2.37917	0.63777	0.92861	1.38517	12.70125
TDEF	4.2262	46.94653	12.69822	0.07411	0.03532	12.4053
TEST	9.3429	6.77701	0.74088	1.05855	0.41925	12.41254
THG1	7.04845	3.95556	0.76354	0.21369	1.99232	13.01342
THG2	5.82148	4.12121	0.8514	0.15381	1.64818	11.89209
THHE	4.61993	6.29074	1.61157	0.16569	0.70261	12.2436
TJOS	6.85692	305.47493	44.28808	0.0101	0.01266	15.5013
TMAN	2.51262	31.53333	15.45772	0.07346	0.03623	12.96793
TMAR	6.57522	29.10753	4.5005	0.06553	0.19565	11.72699
