# Supplementary material for: Testing drivers of acoustic divergence in cicadas (Cicadidae: Tettigettalna)
Source: J Evol Biol. 2022 Dec 13;36(2):461–79. doi: 10.1111/jeb.14133 (PMC10107868; doi:10.1111/jeb.14133)
Supplement: Supplementary file 4 — Table S3 [file JEB-36-461-s003.pdf]

| Variable | Predictor | β ± SE | | | $R^2$ (adj) | p-value |
|---|---|---|---|---|---|---|
| log(NE) | bio1 | 0.042 | ± | 0.020 | 0.207 | 0.058 |
| | bio3 | 0.110 | ± | 0.175 | -0.049 | 0.540 |
| | bio8 | 0.019 | ± | 0.012 | 0.113 | 0.129 |
| | bio9 | -0.007 | ± | 0.030 | -0.079 | 0.829 |
| | bio12 | 0.000 | ± | 0.002 | -0.078 | 0.825 |
| | bio18 | 0.000 | ± | 0.002 | -0.079 | 0.825 |
| log(CD) | bio1 | 0.005 | ± | 0.012 | -0.066 | 0.669 |
| | bio2 | -0.014 | ± | 0.013 | 0.009 | 0.312 |
| | bio3 | 0.021 | ± | 0.096 | -0.079 | 0.824 |
| | bio4 | 0.000 | ± | 0.000 | -0.020 | 0.407 |
| | bio5 | -0.016 | ± | 0.014 | 0.021 | 0.281 |
| | bio8 | 0.002 | ± | 0.007 | 0.725 | -0.072 |
| | bio9 | -0.002 | ± | 0.016 | -0.082 | 0.894 |
| | bio12 | 0.000 | ± | 0.001 | -0.082 | 0.887 |
| | bio18 | 0.002 | ± | 0.007 | -0.072 | 0.727 |
| log(ER) | bio1 | 0.019 | ± | 0.017 | 0.018 | 0.287 |
| | bio2 | -0.025 | ± | 0.019 | 0.054 | 0.212 |
| | bio3 | 0.038 | ± | 0.134 | -0.076 | 0.778 |
| | bio4 | -0.001 | ± | 0.000 | 0.100 | 0.143 |
| | bio5 | -0.020 | ± | 0.019 | 0.013 | 0.301 |
| | bio8 | 0.008 | ± | 0.009 | -0.002 | 0.343 |
| | bio9 | -0.006 | ± | 0.024 | -0.077 | 0.800 |
| | bio12 | 0.000 | ± | 0.002 | -0.082 | 0.914 |
| | bio18 | -0.001 | ± | 0.009 | -0.083 | 0.967 |
| log(ED) | bio1 | -0.016 | ± | 0.017 | -0.012 | 0.374 |
| | bio2 | 0.016 | ± | 0.019 | -0.021 | 0.407 |
| | bio3 | -0.047 | ± | 0.131 | -0.072 | 0.726 |
| | bio4 | 0.000 | ± | 0.000 | 0.036 | 0.247 |
| | bio5 | 0.011 | ± | 0.019 | -0.053 | 0.565 |
| | bio8 | -0.006 | ± | 0.009 | -0.046 | 0.523 |
| | bio9 | 0.006 | ± | 0.023 | -0.078 | 0.810 |
| | bio12 | 0.001 | ± | 0.002 | -0.069 | 0.693 |
| | bio18 | 0.004 | ± | 0.009 | -0.069 | 0.696 |
| log(ID) | bio1 | -0.020 | ± | 0.020 | -0.002 | 0.344 |
| | bio2 | 0.036 | ± | 0.022 | 0.112 | 0.130 |
| | bio3 | -0.028 | ± | 0.161 | -0.081 | 0.866 |
| | bio5 | 0.035 | ± | 0.022 | 0.105 | 0.138 |
| | bio8 | -0.010 | ± | 0.010 | 0.000 | 0.336 |
| | bio9 | 0.014 | ± | 0.028 | -0.063 | 0.637 |
| | bio12 | -0.001 | ± | 0.002 | -0.044 | 0.513 |
| | bio18 | -0.004 | ± | 0.011 | -0.074 | 0.758 |
| log(DF) | bio1 | 0.001 | ± | 0.001 | -0.041 | 0.499 |
| | bio2 | -0.001 | ± | 0.001 | -0.060 | 0.614 |
| | bio3 | -0.007 | ± | 0.008 | -0.010 | 0.371 |
| | bio4 | 0.000 | ± | 0.000 | -0.081 | 0.877 |
| | bio5 | 0.001 | ± | 0.001 | -0.082 | 0.919 |

| | | | | | |
|---|---|---|---|---|---|
| bio8 | 0.001 | ± | 0.001 | -0.022 | 0.412 |
| bio9 | 0.002 | ± | 0.001 | 0.057 | 0.206 |
| bio12 | 0.000 | ± | 0.000 | -0.065 | 0.659 |
| bio18 | 0.000 | ± | 0.001 | -0.058 | 0.604 |
